# Performance analysis of high voltage disc insulators with different profiles in clean and polluted environments using flashover, withstand voltage tests and finite element analysis

**DOI:** 10.1038/s41598-024-71392-5

**Published:** 2024-08-31

**Authors:** Arfan Ali, Abdul Rauf Bhatti, Akhtar Rasool, Fazal Ur Rehman, Muhammad Amjad Khan, Ahmed Ali, Abdulkerim Sherefa

**Affiliations:** 1 Department of Electrical Engineering and Technology, Government College University, Faisalabad, 38000 Pakistan; 2 National Transmission and Despatch Company, Lahore, 54000 Pakistan; 3 Department of Electrical Engineering, University of Botswana, UB0061 Gaborone, Botswana; 4 Department of Electrical and Electronic Engineering Technology, Faculty of Engineering and the Built Environment, University of Johannesburg, Johannesburg, 2092 South Africa; 5 Department of Electrical and Computer Engineering, Wolkite University, 07, Wolkite, Ethiopia

**Keywords:** Power stations, Electrical and electronic engineering, Power distribution

## Abstract

Severe pollution-induced flashovers on insulators present a pressing challenge to power system safety. The frequent failure of high-voltage insulators, particularly in the polluted environments of Pakistan, poses a critical concern. This paper investigates the impact of insulator profile on reducing pollution flashovers, testing two designs as per IEC standard 60383 and simulated using the Finite Element Method in COMSOL Multiphysics®. The test results revealed that deep under-ribs insulators exhibited a 5.008% reduction in flashover voltage, while alternating shed insulators experienced a 3.233% decrease in polluted conditions compared to clean conditions. Notably, under both clean and polluted conditions, alternating shed insulators consistently outperformed deep under-ribs insulators, with a 25.377% higher flashover voltage in clean conditions and a 27.400% superiority in polluted conditions. Computational analysis through the Finite Element Method in COMSOL Multiphysics shows a consistent pattern in potential distribution with increasing insulator count, but the presence of a pollution layer introduces spikes in the electric field distribution, validating experimental results. These findings highlight the superior performance of alternating shed insulators, especially in polluted environments.

## Introduction

The high-voltage transmission network is crucial for transporting electricity from power plants across diverse terrains, including coastal areas, open fields, hilly regions, deserts and forests^1^. A key component of the network is the insulator, which ensures a safe electrical distance from the ground and transmission towers while providing electrical isolation^2^. Transmission utilities worldwide select insulator profiles based on conditions like pollutants, humidity, temperature and local weather across varied terrains^3–6^. A major issue for power utilities worldwide is the failure of outdoor insulators due to flashover, which is primarily caused by industrial, coastal and environmental pollution^7–9^.

Electric transmission networks use various insulators to support and insulate conductors, preventing unintended electrical paths^10,11^. Significant research on high-voltage networks has advanced the understanding of porcelain and glass insulators, emphasizing their electrical resistance and durability under various environmental conditions^12–14^.

For many decades, Porcelain insulators, widely used for their strong electrical and mechanical properties, provide effective insulation by preventing current flow through components^15–18^. They are also used in substation equipment and railway electrification^19^. However, their low hydrophobic properties often result in pollution layers forming on the insulator surface, leading to flashovers and leakage currents in wet conditions^12,20–22^. This can lead to electrical issues like flashovers or tripping in high voltage transmission lines^16,17,23–27^. Additionally, they are brittle and susceptible to damage during transportation or installation^15,28^. Glass insulators, favored for their transparency which aids in detecting damage^29^, can also shatter under stress and face similar pollution issues as porcelain insulators^15,30^. Additionally, they are heavier compared to polymer insulators^28^.

The adoption of polymer insulators in the 1980s marked a significant shift due to their improved hydrophobic properties compared to porcelain and glass insulators^18,31^. The Studies show that polymer insulators reduce the risk of pollution-induced flashovers better than porcelain and glass insulators^13,32^. They are increasingly used in high-voltage transmission lines, especially in polluted areas^21^. However, polymer insulators have limited long-term performance and can degrade under UV radiation, weathering and due to their organic nature^21,32–35^. This deterioration limits their prolonged use in overhead networks^31,36,37^. They are also susceptible to vandalism and bird attacks due to their lighter more flexible nature^27^.

Applying hydrophobic coatings can reduce contamination-related flashovers by preventing water film formation on the insulator surface^20^. Using nanocomposites can enhance the mechanical strength, thermal stability and electrical performance of insulators. The author^38^ concludes that nanodielectrics are advanced materials suitable for electrical power applications^20^. Optimizing shed profiles improves insulator performance in polluted environments by enhancing self-cleaning properties^39^. Developing more flexible insulators can improve mechanical resilience and reduce installation and operation damage risks^40^.

The IEC standard 60,815 categorizes insulator pollution levels from light to very heavy, guiding the selection of insulator profiles and materials based on regional pollution severity^41^.

The design and configuration of insulator profiles significantly impact pollution performance by reducing contaminant accumulation, making profile selection essential for optimal insulation^3,8,9,42–44^. However, the 3-shed profile remains understudied in the context of performance assessment and comparative analyses in polluted environments, indicating a research gap^45^.

The performance of 1512L deep under-ribs insulators has been tested in High Voltage Laboratories worldwide under clean and polluted conditions. Simulation studies also examined the impact of pollution on potential distribution across these insulators, revealing patterns after a certain number in the chain^46^. A study uses COMSOL software to investigate the electrical performance of 11 kV outdoor porcelain and glass deep under-ribs insulators. The analysis shows that contamination results in slightly higher electric fields near the high voltage end of porcelain insulators compared to glass^47^. Another study examines how the ratio of top to bottom surface salt deposit density (SDD) affects the AC pollution flashover performance of disc insulator strings with 2-shed insulators. Results show that decreasing the ratio from 1/1 to 1/15 increased flashover strength by 26%, reflecting the rise in overall pollution layer resistance^48^. Another study experimentally investigates pollution-induced flashover on 175CTV outdoor insulators, widely used by SONELGAZ, under distilled water and sand pollution. Using COMSOL Multiphysics, the research provides congruent and original results, bridging laboratory experiments with computational simulations^49^. However, the 3-shed profile remains understudied in the context of performance assessment and comparative analyses in polluted environments.

The study explores flashover characteristics of high-voltage disc insulators in both clean and polluted conditions, focusing on the effects of environmental factors and aging on insulator performance, including degradation of hydrophobicity and material integrity^15,33,40^. By elucidating these complexities, this work aims to advance the field's knowledge base and provide insights essential for improving design and maintenance strategies of HV transmission systems.

This research proposes an optimized insulation profile for transmission lines in polluted environments by reviewing current designs and evaluating the performance of alternating shed and antifog deep under-ribs insulators. Testing was performed at distinct location along the existing transmission line using withstand voltage and power frequency flashover tests per IEC-60383 criteria. These tests, conducted at the University of Engineering and Technology Lahore-Pakistan, validate insulator performance under clean and polluted conditions.

Further, this study used COMSOL to assess the electric field and potential distribution of both alternating shed and deep under-ribs insulators, with and without a pollution layer. It also analyzed the electric potential-leakage distance graph, providing critical insights into insulator performance using Finite Element Method (FEM) in COMSOL Multiphysics^50,51^. FEM is chosen for its versatility in solving partial differential equations and evaluating electric fields in high-voltage environments^52–54^.

## Site selection for real-world pollutant exposure

**Fig. 1:**
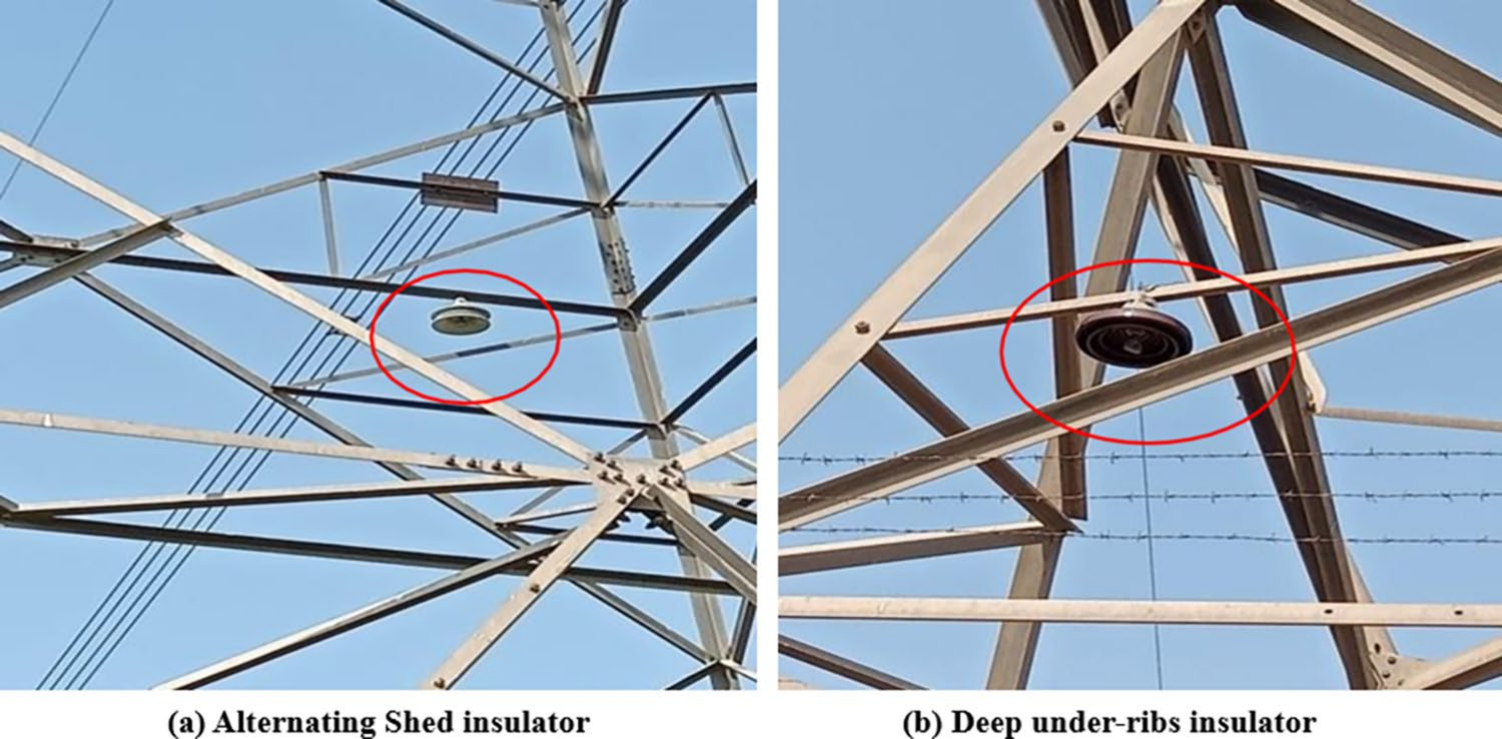
Installation of alternating shed and deep under-ribs porcelain disc insulators at Tower No. 793 of 500 kV West Faisalabad–Gatti transmission line.

To assess the impact of environmental and industrial pollutants on alternating shed and deep under-ribs porcelain disc insulators under un-energized conditions, various surveys/visits were conducted at various locations alongside the existing high voltage transmission lines. To simulate real-world pollutant exposure, one insulator of each type was suspended at Tower No. 793 on the 500 kV West Faisalabad-Gatti transmission line, as depicted in Fig. 1. This specific tower was chosen due to its vulnerability to pollutants, as indicated by historical records of insulator flashovers maintained by the relevant office. Historical records from Transmission Line (Maintenance) Sub Division National Transmission and Despatch Company (NTDC) Faisalabad, Pakistan indicate that this particular tower location is susceptible to flashovers due to factors such as bird droppings, the pollution from nearby brick kiln and industry. The particular location is also prone to other different types of the environmental pollutants due to highly polluted environment of Pakistan, being ranked as one of the top countries in Pollution Index^55^.

**Fig. 2:**
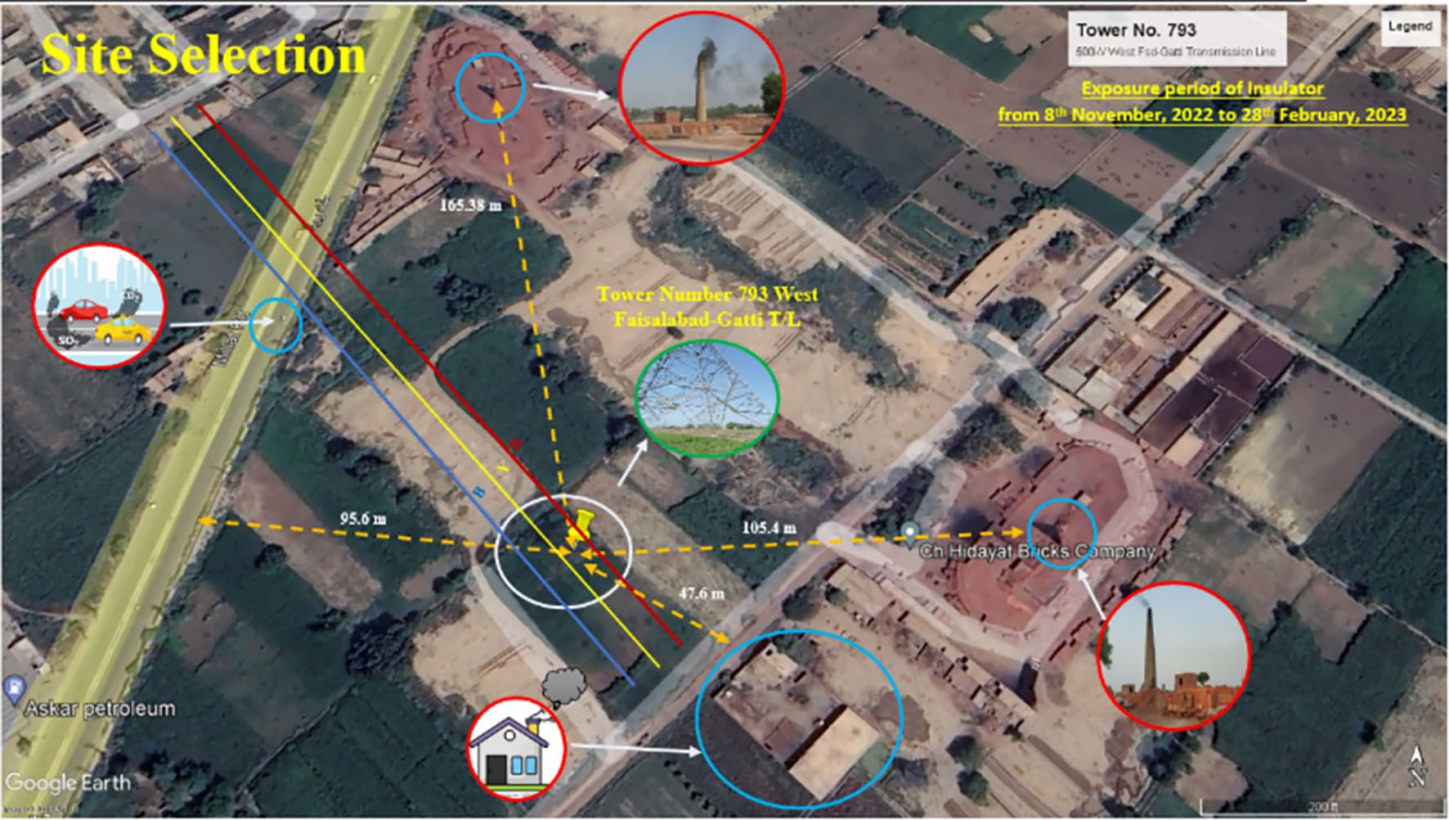
Geographical location of the selected area showing different sources of pollution^58^.

The significant factor in choosing this location is the presence of nearby brick kilns, which frequently release particulate matter, dust, and soot as byproducts of their combustion processes into the atmosphere. The geographical location of the selected area showing different sources of pollution such as brick kilns, highway and domestic chimney, capturned from Google Earth, is shown in Fig. 2. These airborne particles settle on the surface of insulators and establish a conductive pathway, resulting in surface leakage currents. Brick kilns emit a variety of gases during the combustion of diverse fuels and the processing of raw materials. Commonly released gases include carbon dioxide (CO_2_), carbon monoxide (CO), sulfur dioxide (SO_2_), and nitrogen dioxide (NO_2_). These emissions from brick kilns exert adverse effects on the insulating properties of surfaces, leading to heightened leakage currents and an elevated risk of flashovers and insulation breakdown. General Combustion reactions in brick kilns is given below^56,57^.

1 $$Natural Coal + {O}_{2} C{O}_{2} + {H}_{2}O + S{O}_{2} + Heat$$

**Fig. 3:**
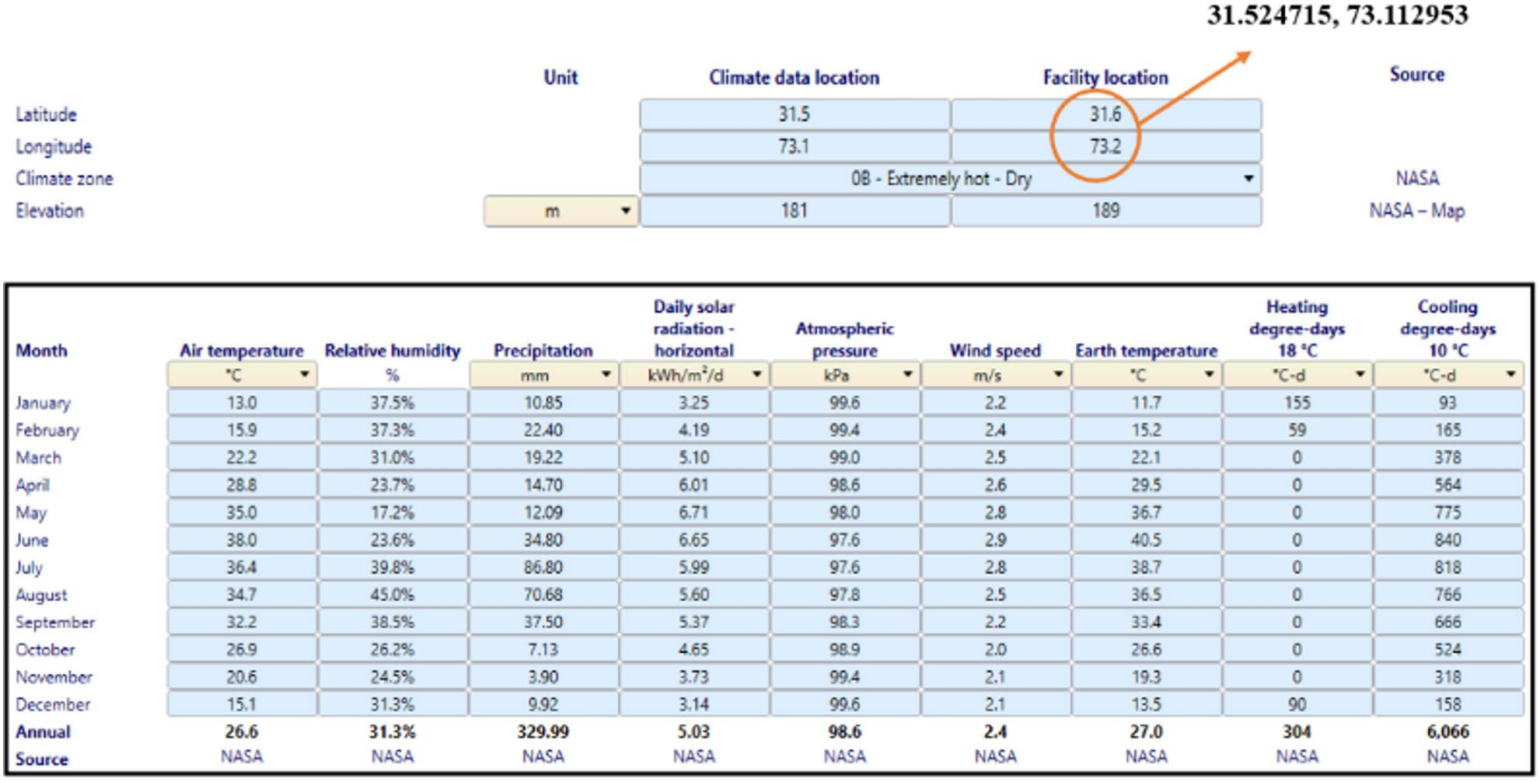
Climate data of the selected location obtained from RETScreen software.

**Fig. 4:**
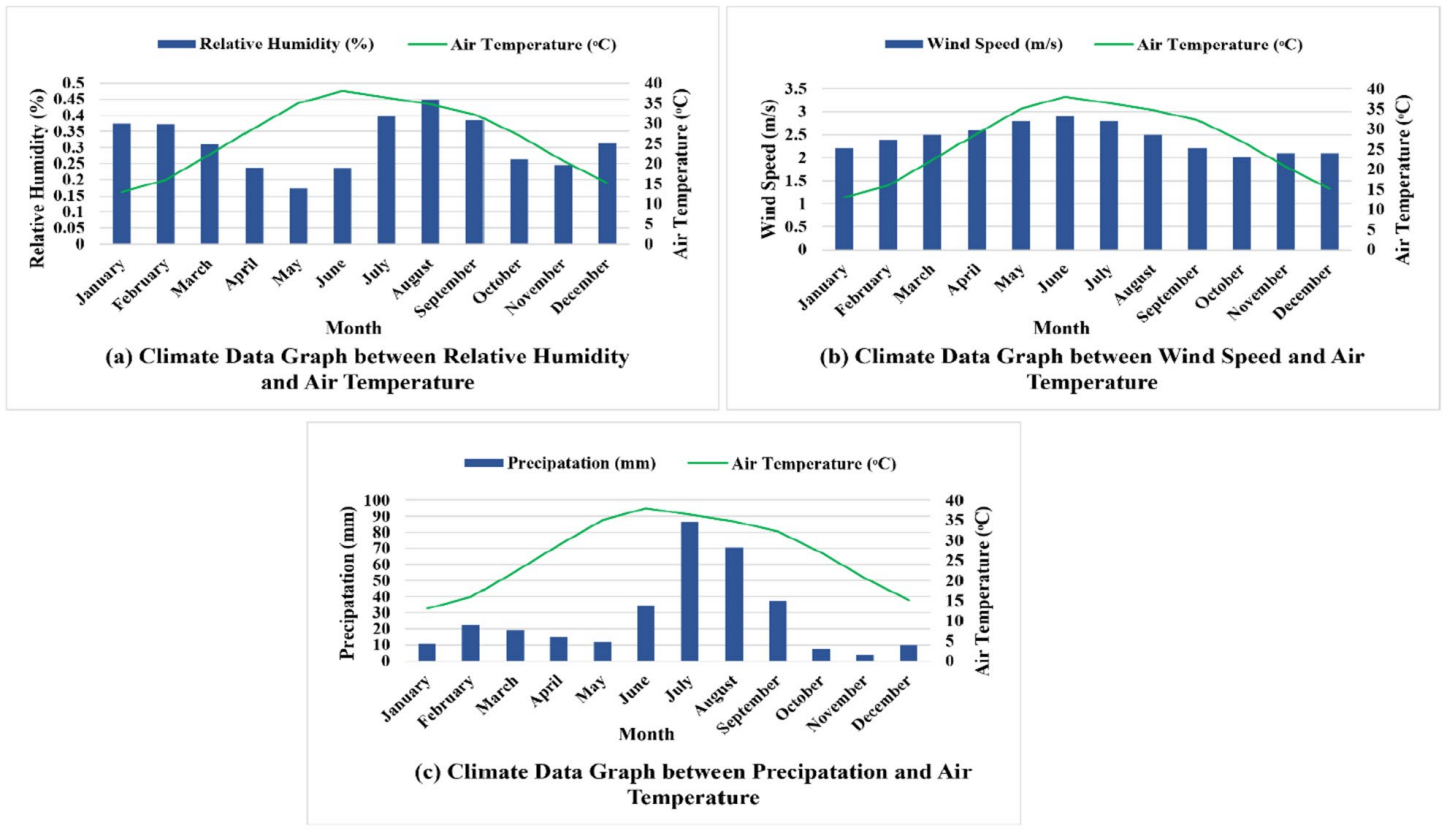
(**a**) Graphical representation of relative humidity, (**b**) wind speed and (**c**) precipitation correspond to air temperature.

High humidity, low wind speed and low precipitation collectively contributes to a conducive environment for flashovers on high-voltage lines. Elevated humidity levels enhance air conductivity, making it easier for electric discharges to occur. Low wind speeds reduce the natural cleaning effect on insulator surfaces, allowing the accumulation of contaminants that compromise insulation. With low precipitation, insulators do not receive adequate cleaning and the absence of moisture can hinder the formation of a conductive layer. These conditions significantly increase the likelihood of flashover incidents, especially in polluted environments. The climate data of the selected location, obtained from NASA's database using RETScreen software, validates that weather from November to February shows high relative humidity, low wind speed and low precipitation, as given below in Fig. 3. Furthermore, the graphical representation of relative humidity, wind speed and precipitation correspond to air temperature is shown in Fig. 4a–c.

Furthermore, the author in paper^59^ carried out a critical study on detection of pollution level of overhead insulators by measuring pH and conductivity of the contaminants of different areas of Faisalabad. Amongst those, one spot is located in the vicinity of our chosen site, i.e. Tower No 793 of 500 kV West-Faisalabad-Gatti transmission line. After investigating the area, the author concluded that higher relative humidity, in addition to a high Equivalent Salt Deposit Density (ESDD), plays an important effect in reducing the flashover voltage which puts the insulator to flashes-over, because the availability of dissociated water ions rises at higher relative humidity. This study, in conjunction with historical tower records and NASA Database data, validates the suitability of the site selection for insulator testing, assessing performance in polluted environments.

## Exposure period of insulator

**Fig. 5:**
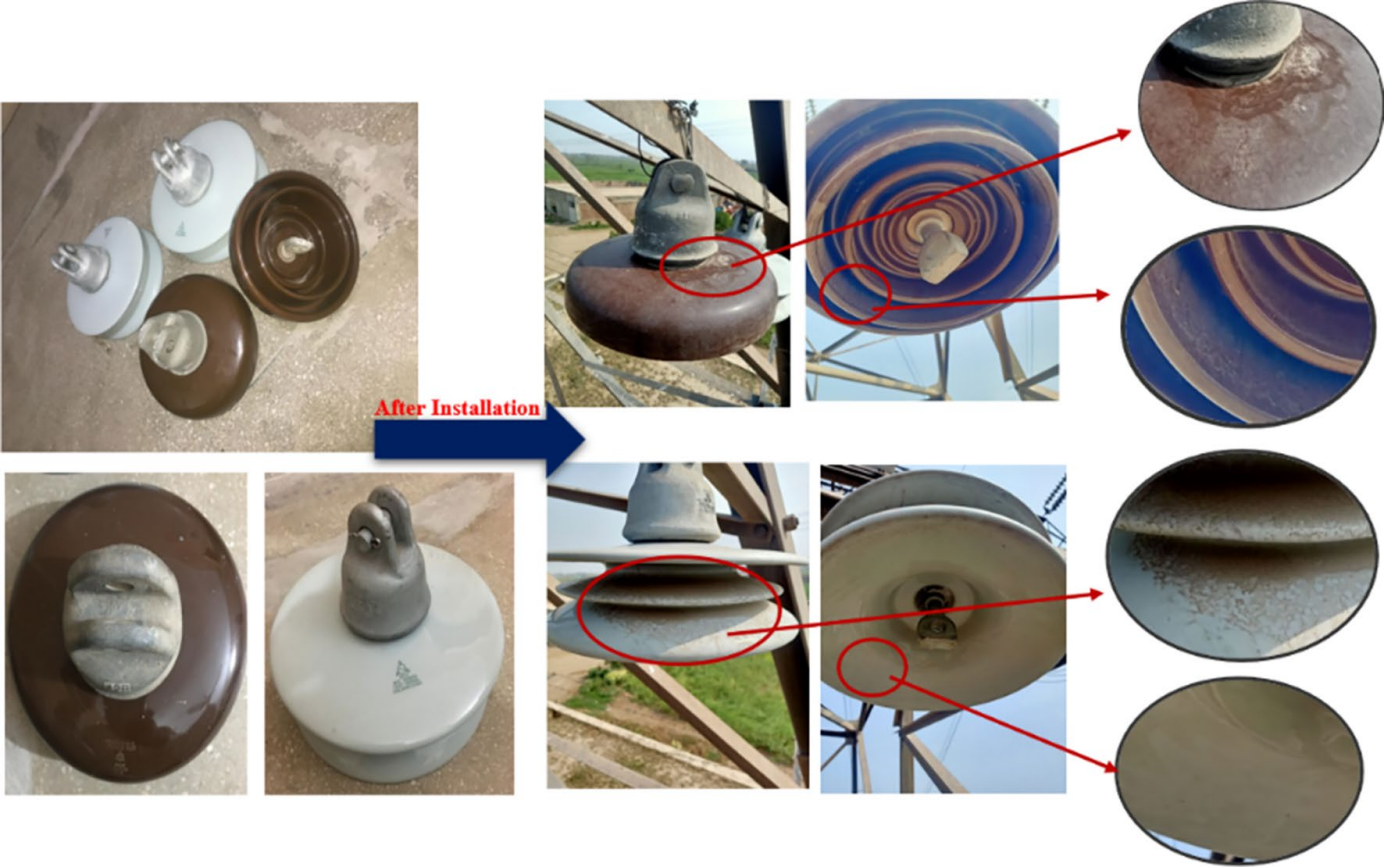
Condition of insulators before and after installing at the tower.

The insulators were exposed at the selected location described above from 8th November, 2022 to 28th February, 2023 for approximately 2457 h. Consequently, this exposure led to the accumulation of a substantial level of pollution on the surfaces of the insulators and the condition of insulators before and after installing at the Tower No 793 of 500 kV West-Faisalabad-Gatti transmission line is shown in Fig. 5.

## Experimental testing

### Creepage test

Creepage test is performed to determine the creepage distance which is an important factor in selection and dimensioning of insulator. A larger creepage distance, also known as leakage distance, prevent accumulation of dust particles, moisture, salt, carbon which cause flashovers and tripping of electric transmission lines. It also increases the path length of current flow and restrain the leakage current, thus minimizing the chance of electrical short circuits or faults.

There are two different test methods for measuring the creepage distance of disc insulators. Before employing any creepage test method, it was ensured that there is no visible damage, crack, or signs of deterioration on the surface of the insulator. After that, dimensions of the disc insulator i.e. circumference of insulator, diameter and spacing between the sheds is measured and creepage distance is calculated by using following formula^60,61^:

2 $$\text{Creepage Distance }= \frac{\text{Circumference of insulator}}{\text{Number of sheds}} +\text{ Spacing between sheds}$$

3 $$\text{Circumference of insulators }=\uppi \times \text{ Diameter}$$

The spacing between sheds provide the gap length between two adjacent sheds of the insulator. By placing values of the number of sheds, spacing and diameter in above formulae, the creepage distances of alternating shed insulator and deep under-ribs insulators are determined which are shown in Table 1.

**Table 1:** Creepage distance of (extra high voltage) EHV insulators.

Sr. no	Insulator profile	Diameter (mm)	Circumference (π × d)	No of sheds	Spacing b/w sheds	Creepage distance
1	160kN Alternating shed	360	1130.4	03	170	546.8
2	160kN Deep under-ribs	390	1224.6	05	185	491.1

**Fig. 6:**
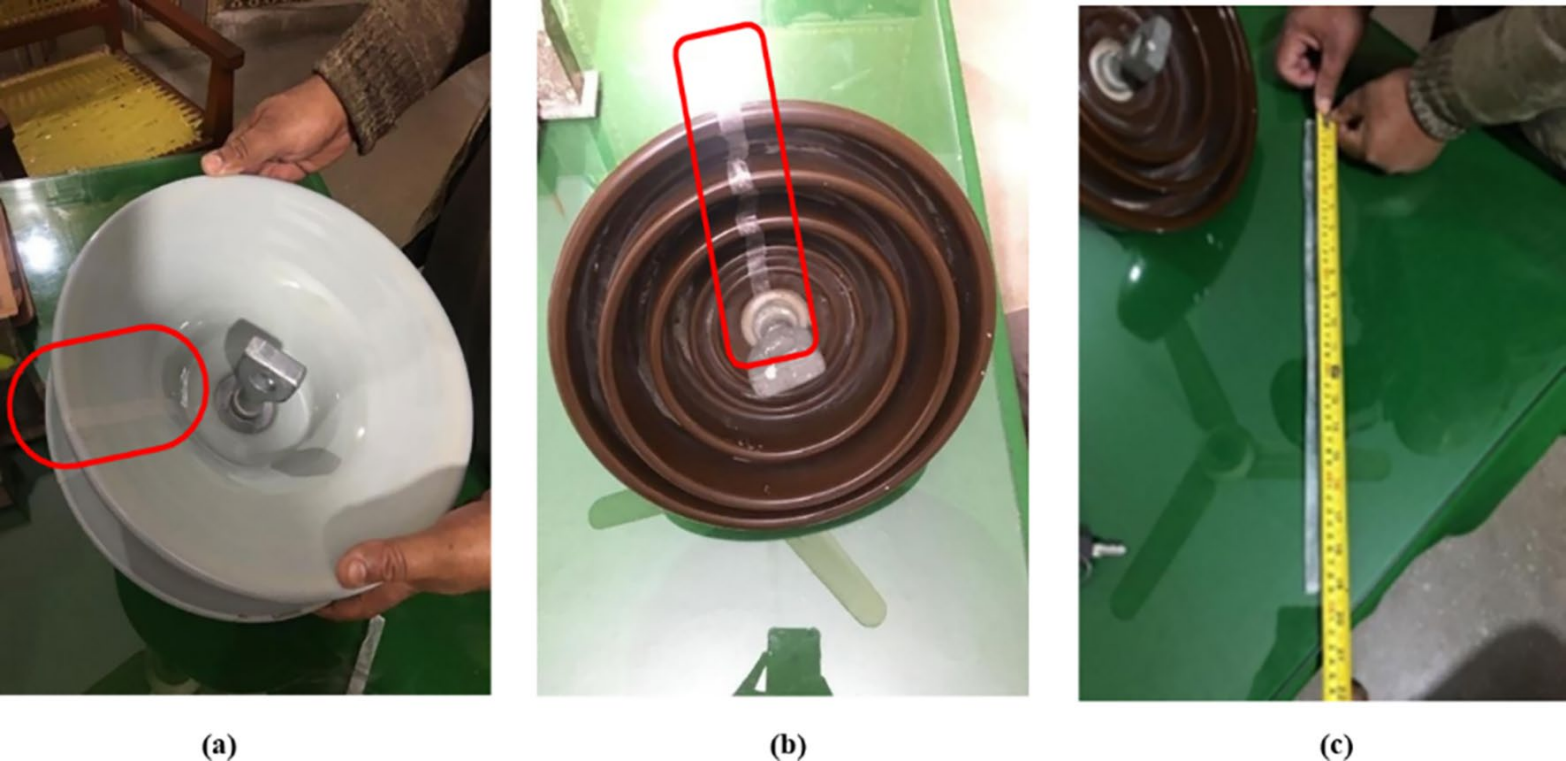
Determining of the creepage distance of alternating shed and deep under- ribs porcelain disc insulators.

In other test method, paper tape and flexible measuring tape is used, as tools for the determination of the creepage length of the disc insulators. To begin with this test, the paper tape is initially wrapped along the path of the sheds around the entire porcelain part of the disc insulators. After that, paper tape is removed from the insulator carefully in such a way that no piece of tape is damaged. Then length of removed piece of paper tape is measured with flexible measuring tape. The measured creepage distances for alternating shed and deep under-rib porcelain disc insulators were found to be approximately 545 mm and 490 mm, respectively. These measurements were taken from the clean surfaces of the insulators, as illustrated in Fig. 6. Despite both insulators possessing similar electromechanical strengths, the creepage distance of the alternating shed insulator exceeds that of the deep under-rib insulator by approximately 40%. A greater creepage distance confers several advantages, including heightened reliability and a reduction in the risks associated with electrical accidents, fires, and equipment damage arising from insulation failure. This outcome underscores the significance of optimizing creepage distances in electrical systems to ensure safety and operational integrity.

### Laboratory testing

The power frequency flashover and withstand voltage test is conducted to determine the voltage level at which flashover occurs across an insulator's surface under power frequency and withstand conditions. The primary objective of these tests was to access the insulators' ability to withstand high voltage levels without experiencing any disruptions or breakdowns. These involved subjecting the high voltage insulators to a withstand voltage test to ascertain their capacity to endure elevated voltage stress, ensuring their reliability under varying environmental conditions. These tests involves two conditions, "clean" and "polluted," and meticulous preparation of the insulator's surface to eliminate defects. Standardized testing procedures International Electrotechnical Commission (IEC)-60,383, are strictly followed to ensure consistent and reliable results.

#### Laboratory test setup

**Fig. 7:**
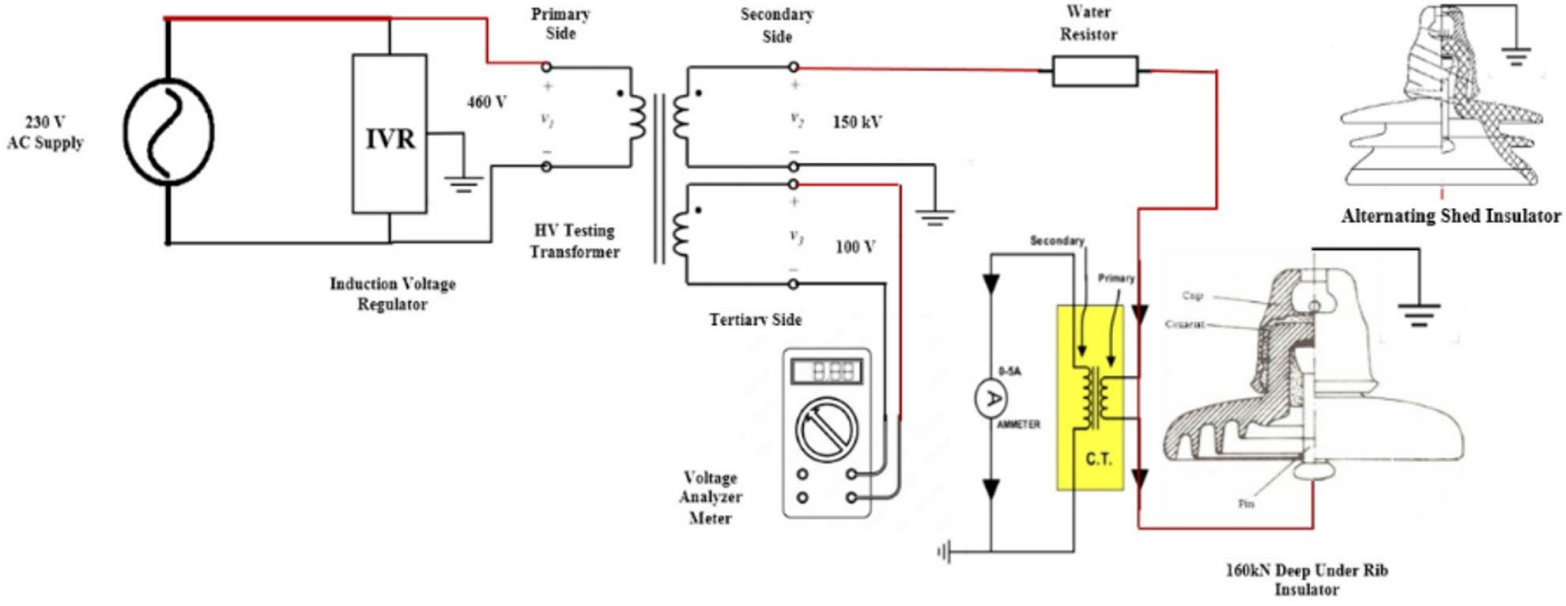
Connection diagram of alternating shed and deep under-ribs insulators.

The performance of both deep under-ribs and alternating shed porcelain insulators is assessed through a power frequency flashover and withstand voltage test, conducted under clean and polluted conditions. To execute this examination, the facility of University of Engineering & Technology (UET) Lahore Pakistan i.e. High Voltage Laboratory was used where, a three-winding, oil-immersed 150 kV alternating current transformer, along with its associated control panel, is employed. The primary and secondary sides of the AC transformer have a rated capacity of 25kVA, while the tertiary side boasts a 50kVA rating. The voltage specifications are as follows: 460 V for the primary side, 150 kV for the secondary side and 100 V for the tertiary side. An induction voltage regulator at 230 V/480 V, in conjunction with an AC voltmeter (ranging from 0-300 V with an accuracy of 1.5), are instrumental in facilitating this test. The connection diagram depicting the arrangement of deep under-ribs and alternating shed insulators with the testing equipment is provided in Fig. 7.

### Power frequency flashover voltage test

#### Test methodology

**Fig. 8:**
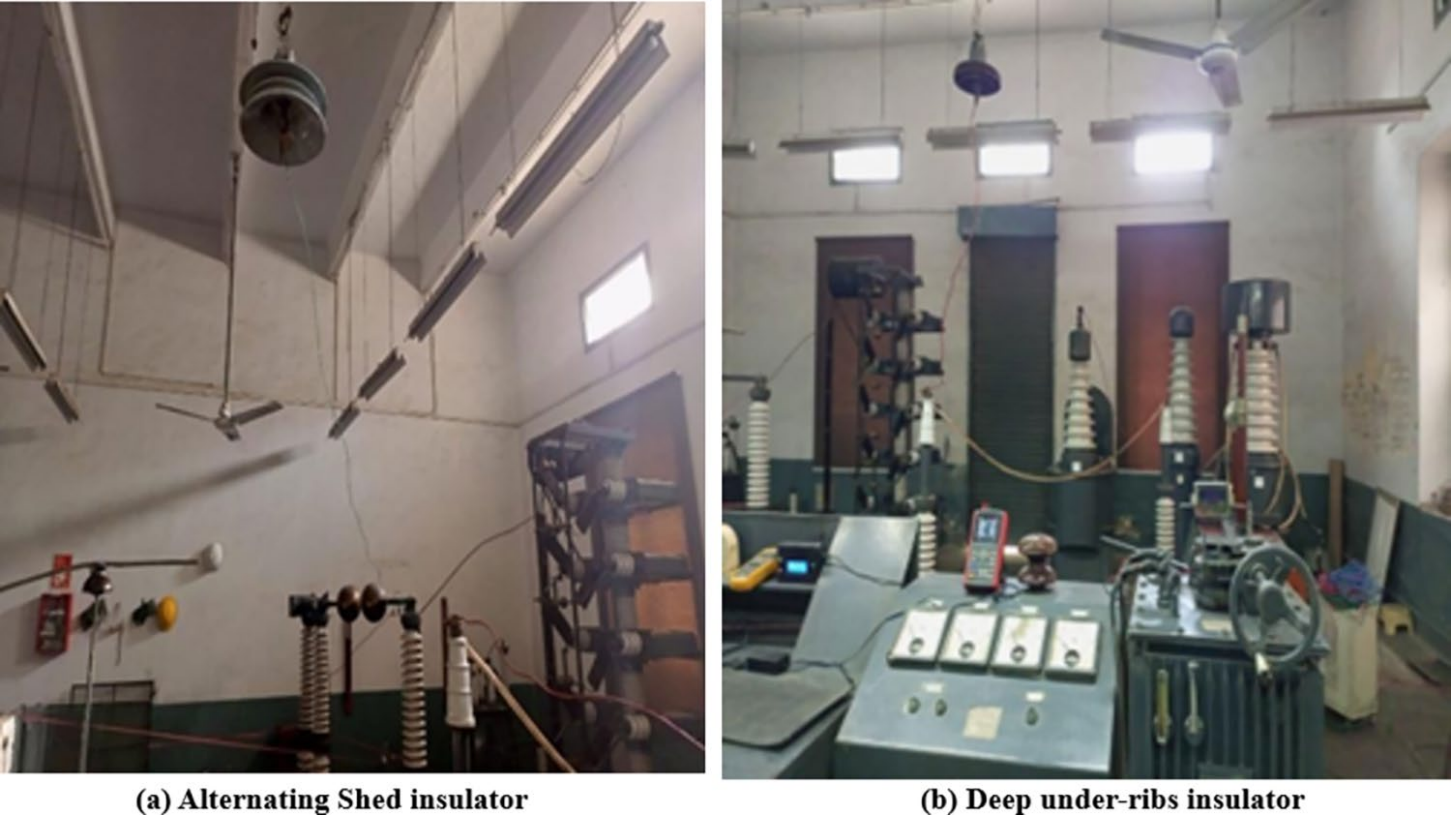
(**a**) Test setup of alternating shed insulator (**b**) Test setup of deep under-ribs insulator.

The power frequency flashover voltage test, conducted in accordance with the international standard International Electrotechnical Commission (IEC-60383), followed a methodical procedure. Prior to commencing the test, the insulators' surfaces underwent thorough cleaning, with the removal of common contaminants such as dust and salt through a combination of water and a universal solvent. Hand wiping further ensured the elimination of contaminants. The cleaning process was exclusively applied to reference insulators, not to those polluted at specific sites. Clean insulators were employed for a comparative assessment alongside polluted insulators to evaluate their performance. Later, the insulators' health was verified using an insulation tester, which indicated the maximum resistance on the meter, confirming their sound condition. Environmental conditions, including room temperature, atmospheric pressure, and humidity, were duly noted. Subsequently, both cleaned and polluted insulators were individually suspended using hooks at a height of 10 feet above ground level. High voltage was applied at the lower end (pin) of the insulator via a connection to the high voltage testing transformer, while the upper end (cap) was securely grounded, following the configuration outlined in Fig. 8. This meticulous process ensured precise testing conditions and accurate evaluation.

Voltage was incrementally adjusted at a rate of about 2 kV per second using a voltage regulator. Simultaneously, the leakage current was monitored and measured intermittently with an ammeter connected to a current transformer (CT). As the voltage increased, it led to the audible and then visible corona discharge, with the corresponding leakage current duly recorded. This methodical procedure facilitated the systematic observation and documentation of corona events in response to voltage variations.

#### Power frequency flashover voltage test results

##### Audible and visible corona

During the Power frequency flashover test under clean conditions, the initial voltages for deep under-ribs insulator, at which audible and visible corona were observed stood at 30 kV and 75 kV, with corresponding leakage currents of 0.174 mA and 0.296 mA, respectively. Whereas, alternating shed insulator exhibited values of 22.49 kV for audible corona and 72 kV for visible corona, with leakage currents of 0.079 mA and 0.304 mA.

While performing Power Frequency Flashover Test on polluted deep under-ribs insulator under same humidity, temperature and other room conditions, audible corona voltage and its corresponding leakage current was found 19.54 kV and 0.761 mA respectively. Whereas, the visible corona voltage was approximately 67.59 kV and its corresponding leakage current 2.429 mA for deep under-rib insulator. On the other hand, the audible corona voltage was approximately 20.3 kV and its leakage current 0.479 mA in the case of alternating shed porcelain disc insulator under polluted condition. Accordingly, the value of visible corona voltage is found approximately 70.63 kV and its leakage current 1.783 mA for alternating shed insulator under polluted conditions as shown in Table 2.

**Table 2:** Audible and visible corona voltage of deep under-ribs and alternating shed insulator under clean and polluted conditions.

Test name	Type of insulator	Audible corona corresponding leakage current		Visible corona corresponding leakage current	
		Clean insulator	Polluted insulator	Clean insulator	Polluted insulator
Power frequency flashover test	Deep under-ribs insulator	30.0 kV 0.174 mA	19.54 kV 0.761 mA	75 kV 0.296 mA	67.59 kV 2.429 mA
	Alternating shed insulator	22.49 kV 0.079 mA	20.3 kV 0.479 mA	72 kV 0.304 mA	70.63 kV 1.783 mA

##### Flashover voltage

**Fig. 9:**
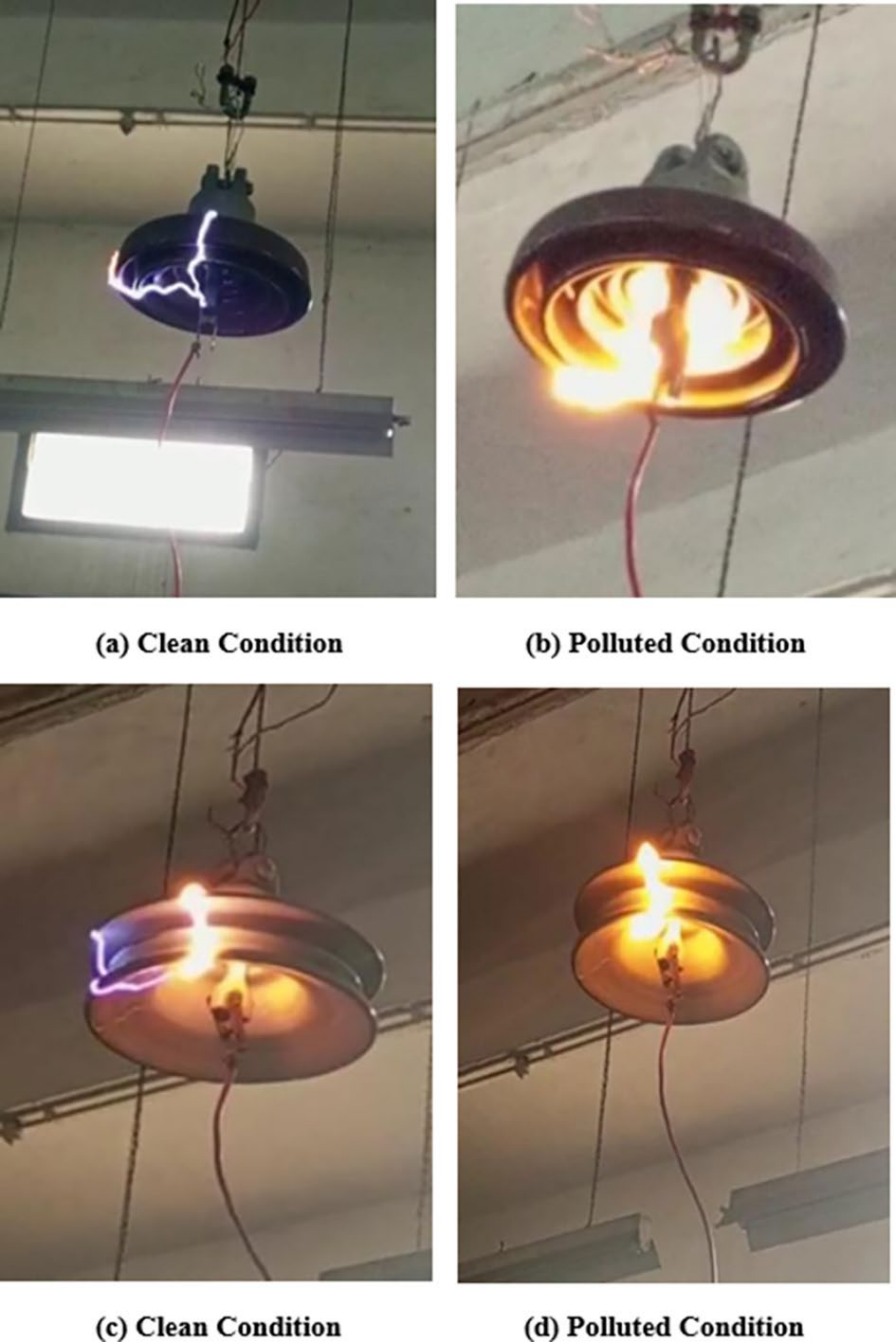
(**a**,**b**) Flashover across deep under-ribs insulator under clean and polluted condition, (**c**,**d**) Flashover across alternating shed insulator clean and polluted condition.

As the applied voltage was gradually increased after observing visible corona, a specific threshold was reached at which flashover occurred across the insulators, as illustrated in Fig. 9.

Under clean conditions, deep under-ribs insulators had an average flashover voltage of 106.08 kV and a leakage current of 2.920 mA just before flashover. On the other hand, alternating shed insulators had an average flashover voltage of 133 kV and a leakage current of 2.606 mA as shown in Table 3. Under polluted conditions, the average flashover voltage of deep under rib insulator came out 101.02 kV and leakage current 3.736 mA just before flashover. Whereas, the average flashover voltage of alternating shed insulator came out 128.7 kV and its corresponding leakage current 3.557 mA just before flashover as shown in Table 3.

**Table 3:** Power frequency flashover test results of deep under-ribs and alternating shed insulator under clean and polluted conditions.

Test name	Type of insulator	Flashover voltage and corresponding leakage current	
		Clean insulator	Polluted insulator
Power frequency flashover test	Deep under-ribs insulator	106.08 kV 2.920 mA	101.02 kV 3.736 mA
	Alternating shed insulator	133 kV 2.606 mA	128.7 kV 3.557 mA

##### Graphical representation

**Fig. 10:**
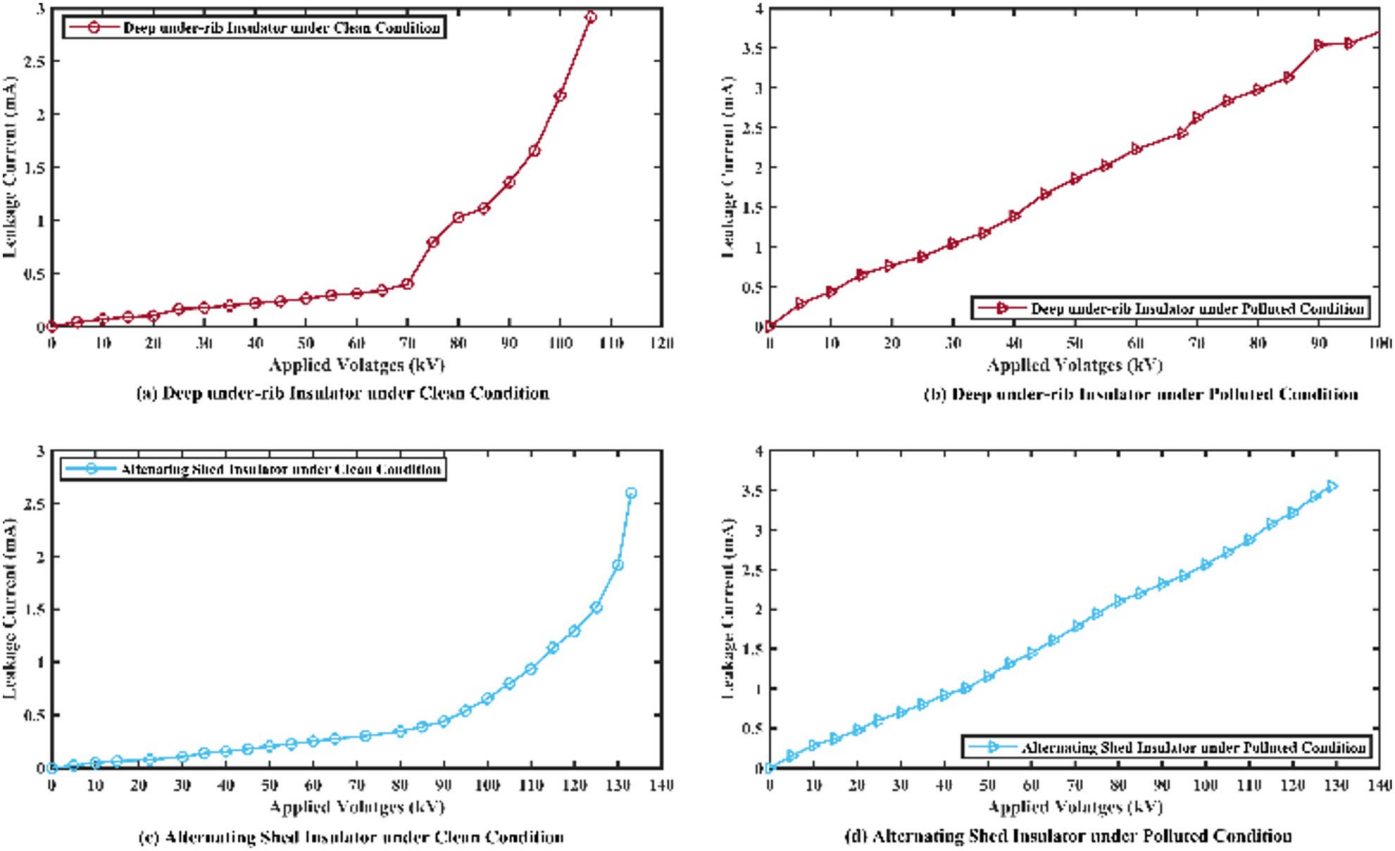
(**a**,**c**) Flashover voltage graph of deep under-ribs and alternating shed porcelain disc insulator under clean condition, (**b**,**d**) under polluted conditions respectively.

The graphical analysis of flashover voltage and its corresponding leakage current of both insulators are shown in Fig. 10. In the graph, the value of x (x-axis) indicates the applied voltages and value of y (y-axis) indicates the corresponding leakage currents and brown line shows the behaviors of deep under-ribs insulator while the sky blue line shows the behaviors of alternating shed insulator.

#### Withstand voltage test

As per testing procedure discussed above in para 4.1.1, A withstand voltage test was performed on deep under-ribs and alternating shed insulators following International Electrotechnical Commission (IEC-60383) standards.

##### Withstand voltage test results

To evaluate the insulators' performance, one-minute duration tests were conducted at voltage levels of 90 kV, 95 kV, and 100 kV for deep under-ribs insulators, and 120 kV, 125 kV, and 130 kV for alternating shed insulators under clean conditions. For polluted conditions, similar tests were performed at voltage levels of 90 kV, 95 kV, and 97.5 kV for deep under-ribs insulators, and at 115 kV, 120 kV, and 125 kV for alternating shed insulators showing no flashover or abnormalities. The insulator's performance was evaluated during this time and no flashover or abnormalities were observed while maintaining the specified voltage levels for one minute as shown in Table 4.

**Table 4:** Withstand voltage test results of deep under-ribs and alternating shed insulator under clean and polluted conditions.

Test name	Type of insulator	Withstand voltages for one minute duration					
		Clean insulator			Polluted insulator		
Withstand Voltage Test as per (IEC-60383–1)	Deep under-ribs insulator	90 kV	95 kV	100 kV	90 kV	95 kV	97.5 kV
	Alternating shed insulator	120 kV	125 kV	130 kV	115 kV	120 kV	125 kV

##### Graphical representation

**Fig. 11:**
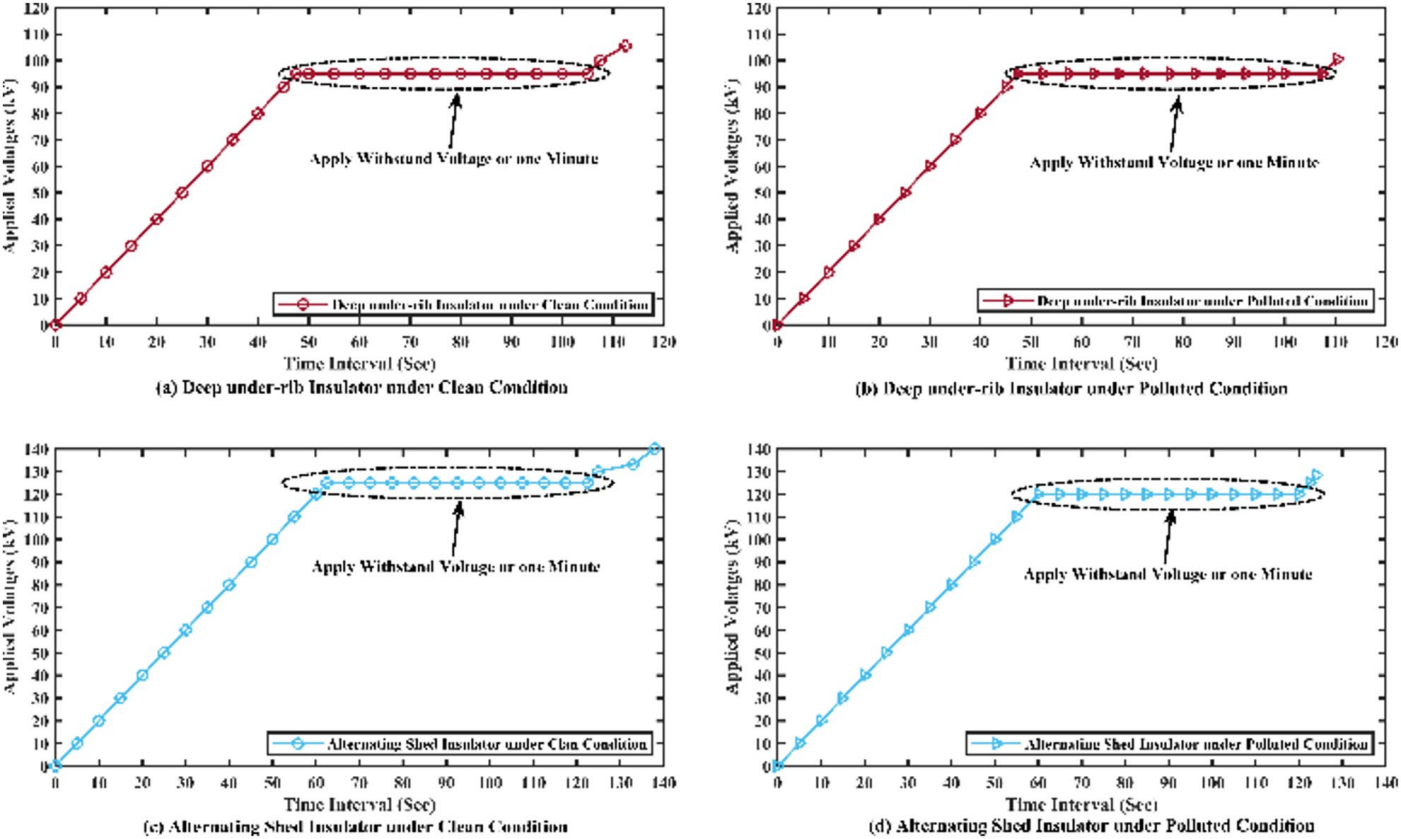
(**a**,**c**) Withstand Voltage graph of deep under-ribs and alternating shed porcelain disc insulator under clean conditions, (**b**,**d**) under polluted conditions respectively.

The results were subsequently presented graphically using MATLAB software as shown in Fig. 11. In the graph, brown line shows the behaviors of deep under-ribs insulator while the sky blue line shows the behaviors of alternating shed insulator.

## Insulators profiles and computational modeling

### Design of the model

In this paper, 2D models of cap and pin deep under-ribs and alternating shed porcelain disc insulators are designed in AutoCAD, initially in the form of singular insulator with and without pollution layer. Then, different strings are formed comprising on different numbers of insulators.

### Insulators profiles

**Fig. 12:**
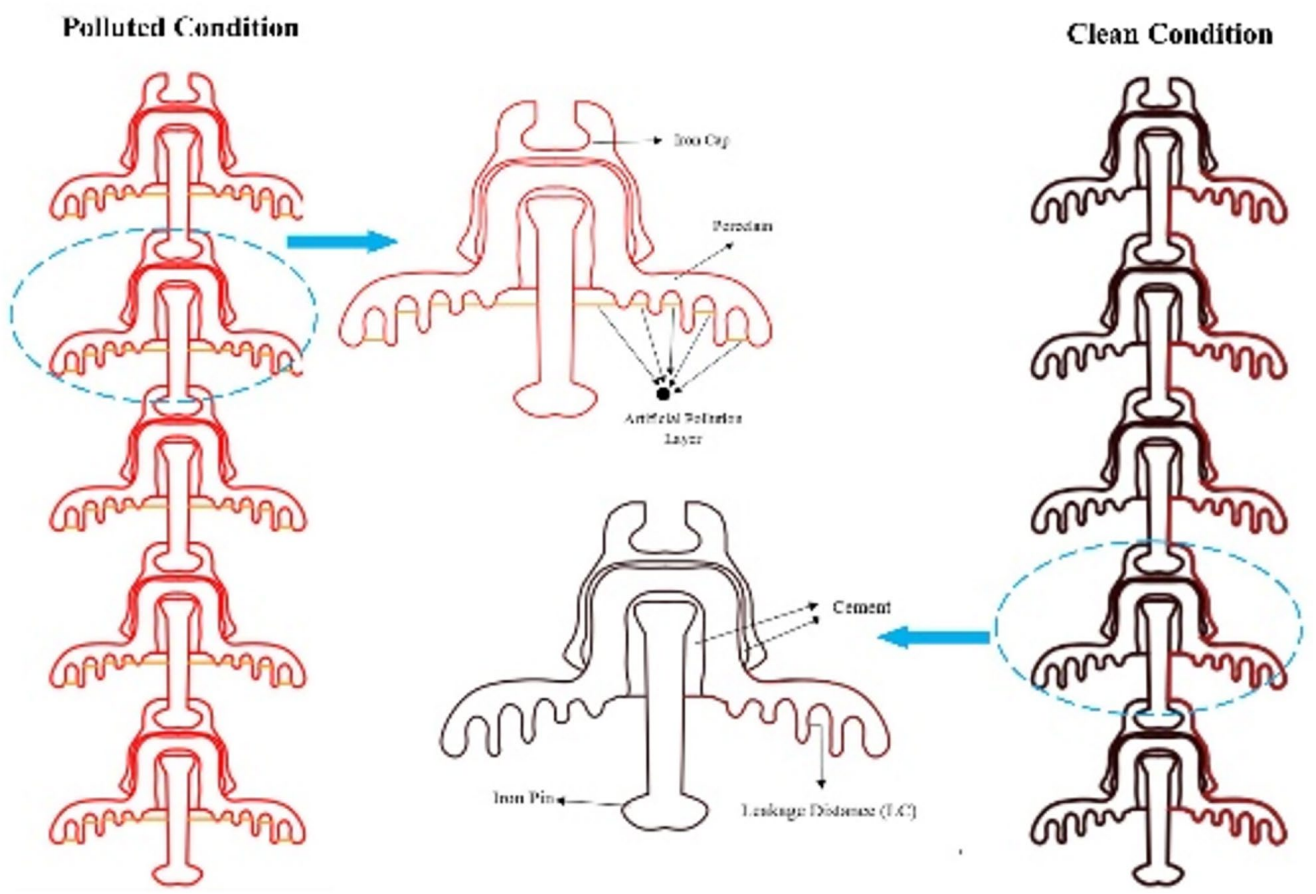
Geometry design of deep under-ribs insulator with and without pollution layer.

**Fig. 13:**
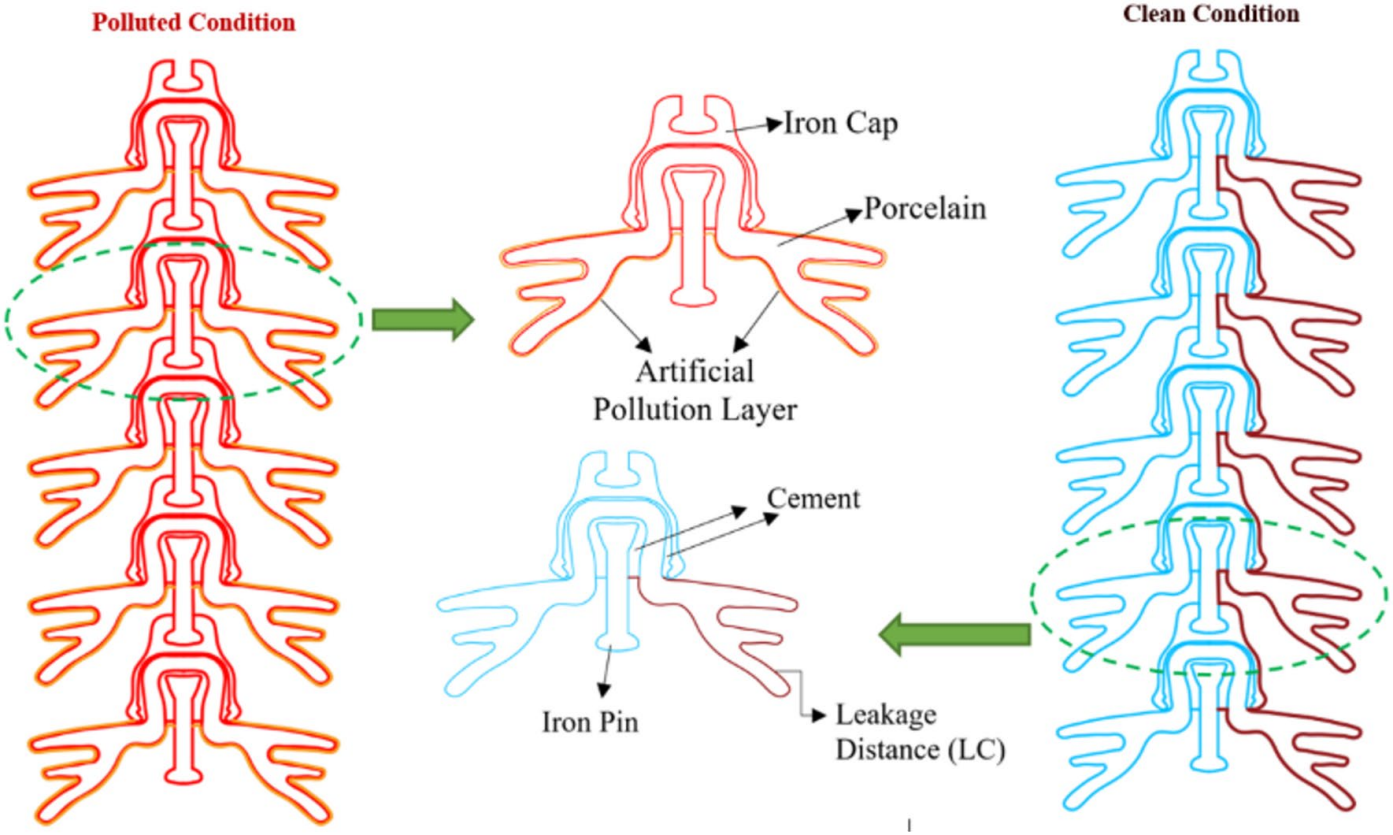
Geometry design of alternating shed insulator with and without pollution layer.

2D wireframe of deep-under-ribs and alternating shed disc insulator was created with the help of AutoCAD software as shown in Figs. 12 and 13.

After that, designed geometries of both types of insulators are imported in COMSOL Multiphysics, which is capable to solve complex problem easily. The uniform thickness of pollution layer is also modeled and stationed near the pin of porcelain insulators. Two boundaries have been established for the models: the first boundary corresponds to the iron pin, where a high electric potential of 25 kV is initially applied for a single insulator. This potential increases by increments of 25 kV for each additional insulator in the series^51^. The second boundary is represented to the iron cap of the last insulator, which is consistently set to a zero voltage level, signifying ground potential, as illustrated in Table 5^51^.

**Table 5:** Electrical potential across each insulators.

Nos. of insulators	Applied electrical potential
One insulator	25 kV
Two insulator	50 kV
Three insulator	75 kV
Four insulator	100 kV
Five insulator	125 kV

These insulators comprise of four different materials i.e. Iron (cap and pin), porcelain (disc & sheds), cement (at the point of attachments of porcelain with cap & pin) and pollution layer. The thickness of pollution layer is 1.5 mm. Relative permittivity of each material/component of the insulators are shown in Table 6^51^.

**Table 6:** Relative permittivity of the different materials.

Materials	Relative permittivity
Iron (pin and cap)	10^6^
Porcelain	6
Portland and cement	5.9
Air	1.0006
Artificial pollution	80

In COMSOL Multiphysics, electrostatics physics is used to study the effect of electrical potential and electric field in clean and polluted conditions. In Finite Element Method (FEM) modeling, boundary conditions were crucial for defining how the model interacted with its environment and ensuring that the problem was well-posed. Several types of boundary conditions, such as Dirichlet Boundary Condition, Neumann Boundary Condition, Periodic, and Symmetric boundary conditions etc. each served different purposes in simulations. A Dirichlet boundary condition, also known as a voltage or potential boundary condition in the context of electric fields, specified a fixed electric potential on a boundary. This is used to set known voltages at surfaces or points, such as grounding a conductor or applying a specific voltage to electrodes. Mathematically, it was expressed as V = V0 on the boundary, where V was the electric potential and V0 was the prescribed value. Electrostatics physics in allows only relative permittivity to calculate the electrical potential and electric field distribution^50–52^.

### Mathematical model

To streamline the computation of the electric field, COMSOL employs the derivative of the electric potential (V), which is described by the following field potential equation^62^:

4 $$E=-\text{grad }V$$

Obtaining Poisson’s equation is exceedingly simple:

Maxwell’s Equation are:

5 $$\nabla .D=\rho$$

The definition of D

6 $$D=\varepsilon E$$

And the gradient relation from Eq. (1):

7 $$E=-\nabla V$$

By substitution Eq. (3 and 4) in 2, we have:

8 $$\nabla .\left(\varepsilon E\right)=\nabla .\left(\varepsilon \left(-\nabla V\right)\right)=-\nabla .\left(\varepsilon \nabla V \right)=\rho$$

or:

9 $$\varepsilon \nabla .\left(\nabla \text{V}\right)=-\uprho$$

Without space charge $\rho =0,$ Poisson’s equation becomes Laplace’s equation:

10 $$\varepsilon \nabla .\left(\nabla \text{V}\right)=0$$

Equation (7) represents the conclusive solver used to calculate the distribution of electrical potential 'V' across a user-defined domain, incorporating source and boundary conditions.

The simulation process involves defining the system's geometry, specifying the relevant physical principles, boundary conditions, and material properties, and setting up the governing equations. After meshing the geometry, the software numerically solves these equations, providing insights into the system's behavior.

### Finite element method (FEM)

In COMSOL Multiphysics, FEM is employed to discretize the geometry into small elements or finite elements, allowing complex problems to be approximated by simpler equations over these elements. In COMSOL Multiphysics, 2D geometry is typically meshed using triangular or quadrilateral elements, depending on the factors like geometry complexity, accuracy requirements, and computational efficiency. Both types of elements can be used to discretize the geometry and facilitate the finite element analysis. Triangular elements are more versatile for irregular shapes, while quadrilateral elements suit structured geometries. To specify the mesh element size in COMSOL, a predefined mesh type “Extra Fine” was chosen for high accuracy and reliability of the simulation results.

Tables 7 and 8 shows the numbers of mesh elements in triangular, edge and vortex region of all models of deep under-ribs and alternating shed insulator respectively in the presence and absence of pollution layer. From this table, it is found that mesh elements increases with increase in the number of insulators.

**Table 7:** No of mesh elements in each deep under rib insulator models.

No of insulators		Without pollution	With pollution
One insulators	Triangle	14,750	22,447
	Edge	982	1826
	Vortex	246	336
Two insulators	Triangle	26,830	42,279
	Edge	1905	3599
	Vortex	475	657
Three insulators	Triangle	39,554	63,117
	Edge	2819	5384
	Vortex	704	978
Four insulators	Triangle	52,688	84,426
	Edge	3726	7156
	Vortex	933	1299
Five insulators	Triangle	65,278	104,903
	Edge	4625	8905
	Vortex	1162	1620

**Table 8:** No of mesh elements in each alternating shed insulator models.

No of insulators		Without pollution	With pollution
One insulators	Triangle	15,132	20,129
	Edge	1000	1237
	Vortex	262	280
Two insulators	Triangle	28,789	37,998
	Edge	1880	2314
	Vortex	491	527
Three insulators	Triangle	41,838	55,002
	Edge	2742	3385
	Vortex	722	776
Four insulators	Triangle	54,499	71,702
	Edge	3592	4422
	Vortex	949	1021
Five insulators	Triangle	67,897	89,233
	Edge	4473	5508
	Vortex	1180	1270

**Fig. 14:**
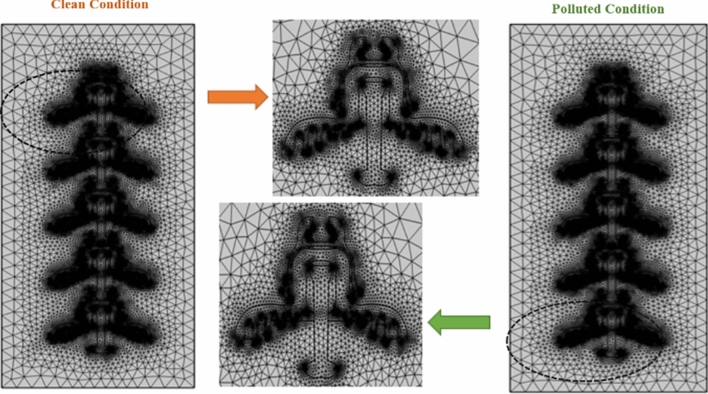
Triangular mesh model of deep under ribs insulators with and without pollution layer.

**Fig. 15:**
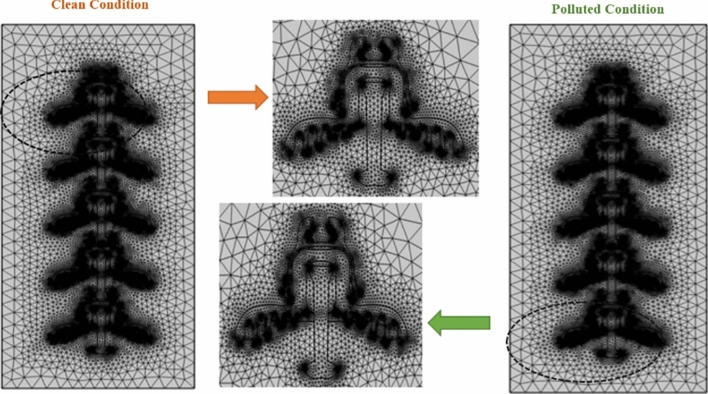
Triangular mesh model of alternating shed insulator with and without pollution layer.

A greater number of mesh elements typically leads to higher accuracy in results, allowing better capture complex geometries and sharp gradients^51^. The triangular meshing of designed models of deep under ribs insulator and alternating shed insulator respectively in clean and polluted conditions are shown in Figs. 14 and 15.

### Simulation results

#### Electric potential distribution

**Fig. 16:**
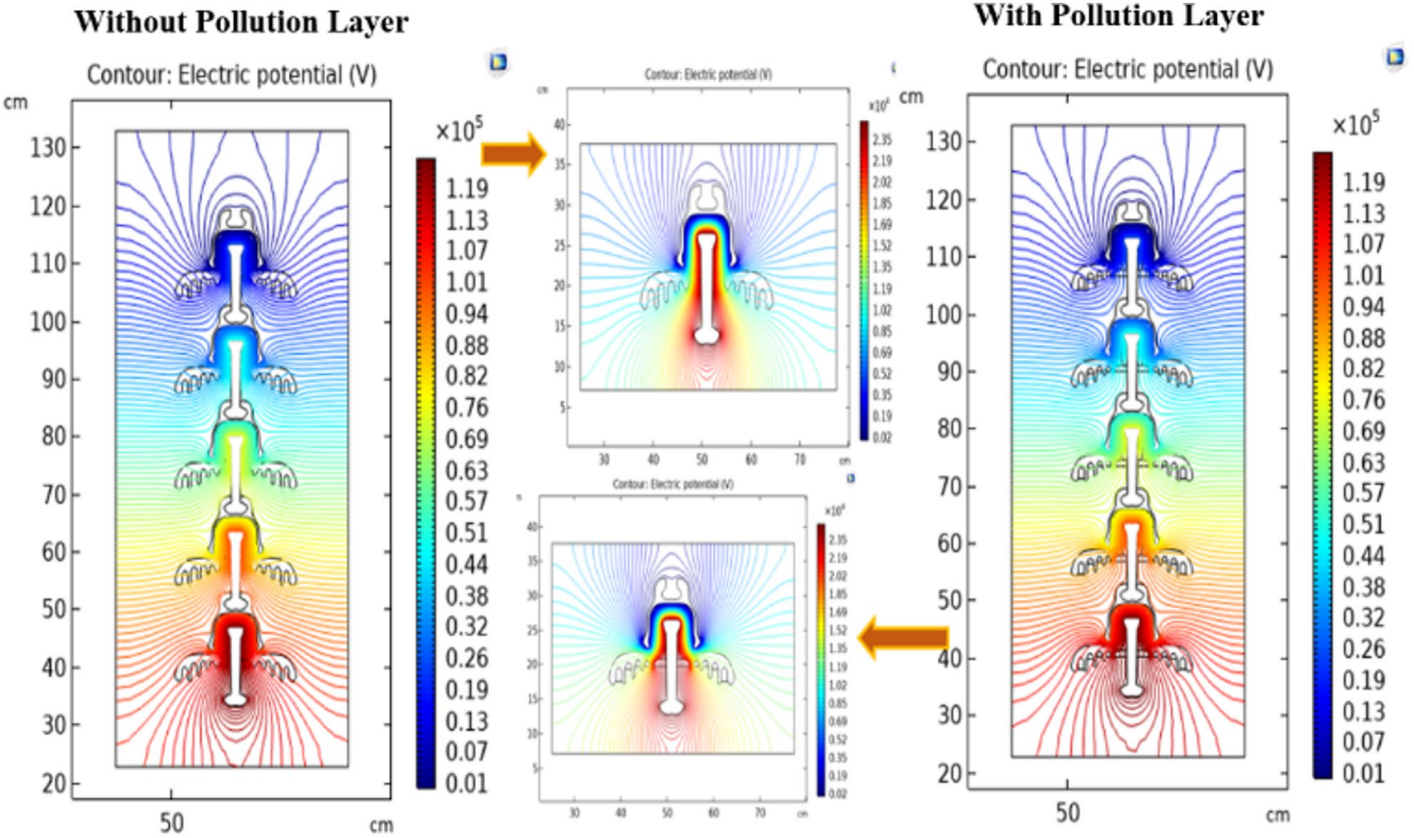
Potential distribution of deep under ribs insulators with and without pollution layer.

**Fig. 17:**
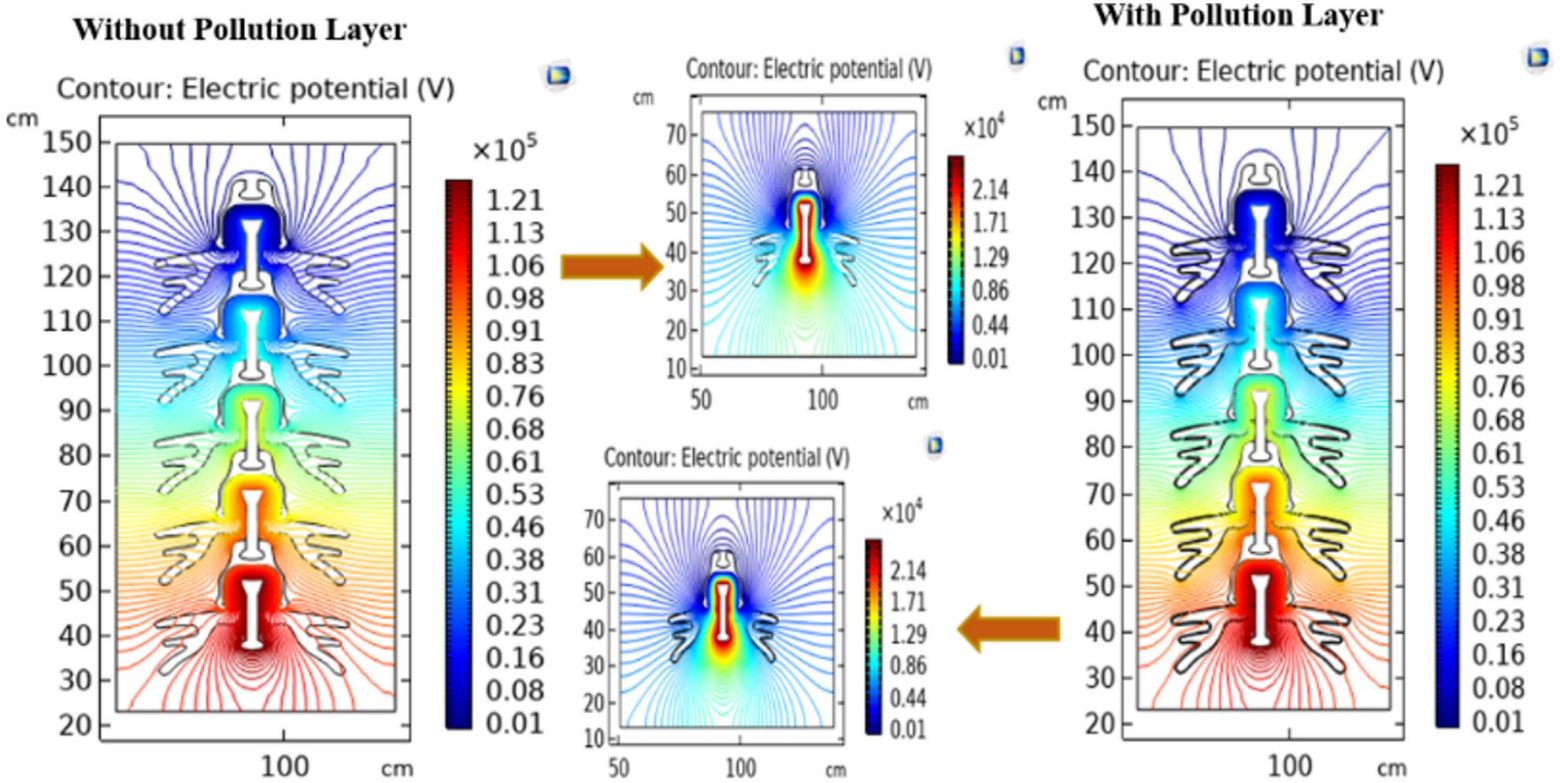
Potential distribution of alternating shed insulators with and without pollution layer.

The electrical potential distributions across different models of the deep under-ribs and alternating shed insulators in clean and polluted conditions are shown in Figs. 16 and 17 respectively. Electrical potential across the insulator’s surface is calculated from pin to the cap of insulator. Contour plots depicting the distribution of electric potential across deep under-ribs and alternating shed insulators are generated for single insulators as well as various combinations of insulators arranged in strings. Figure 16 illustrates these contour plots, including equipotential lines. The results reveal that within the cap and pin of each insulator in the string, there are no contour lines. This absence is attributed to the fact that there is no electric potential difference in the metal components of the insulators.

Conversely, the most significant variations in potential are observed in the non-metallic parts, specifically the porcelain surface and cement. This phenomenon occurs because the electric potential concentrates along the shortest path of the insulator, essentially through the center of the insulator string. However, variations in potential values exist due to the presence of different materials, as clearly demonstrated in Figs. 16 and 17.

As additional insulators are added to the string, there is a drop in potential, evident by changes in the color of the contour lines within these obstructing materials, ranging from the high potential pin to the ground cap. It's important to note that the presence of pollution on the insulator does not distort the shape of the potential distribution. Instead, it alters the concentration of equipotential lines due to the presence of pollution layers^51^.

**Fig. 18:**
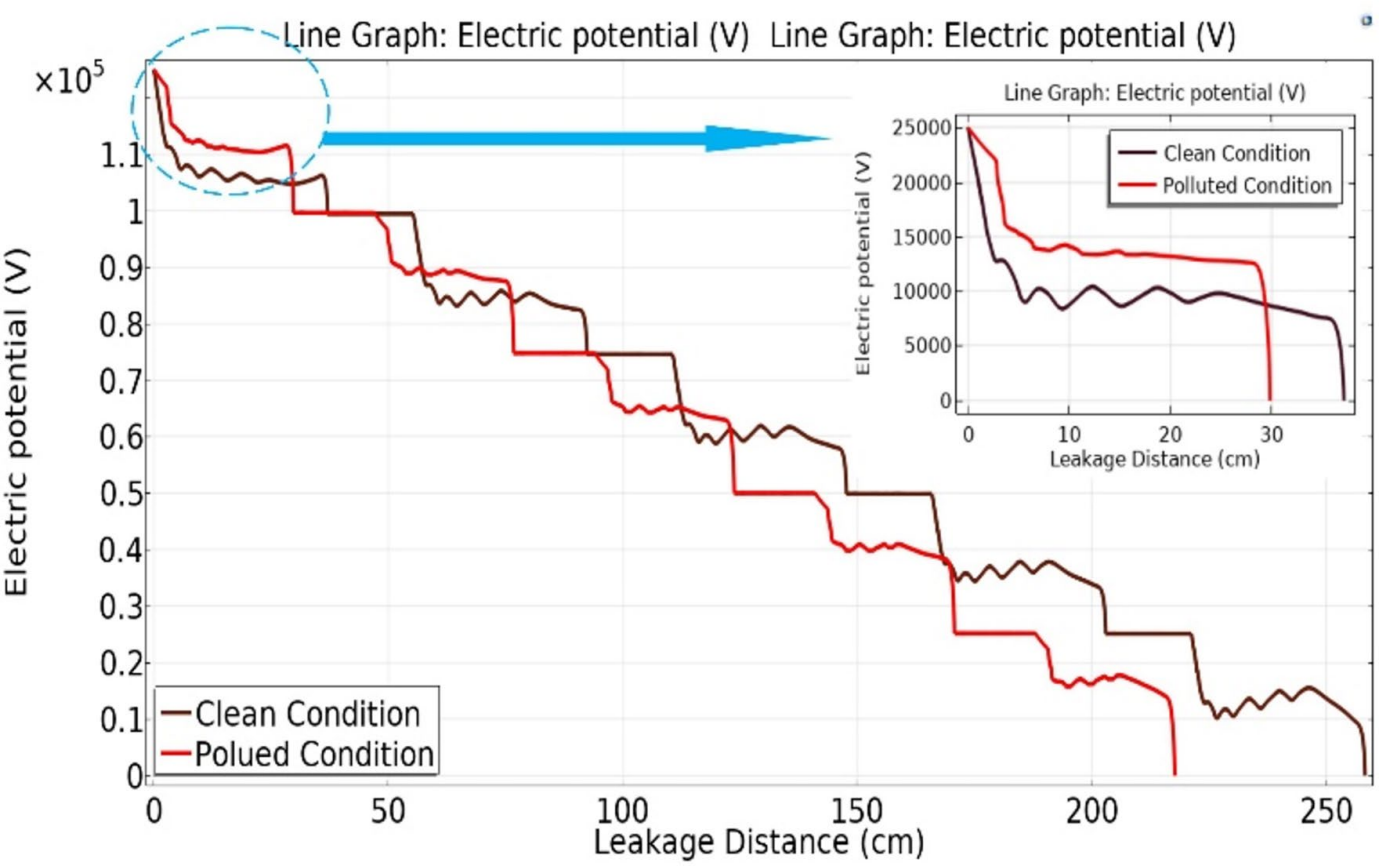
Electric potential-leakage distance graph across deep under-ribs insulators.

Figure 18 shows the variation in electric potential relative to the leakage distance for different models of deep under-ribs insulator strings, encompassing five insulators. A noteworthy observation from this figure is that, in the presence of pollution, the potential values are significantly higher when compared to clean conditions. Examining Fig. 18, it becomes apparent that as the number of insulators in the chain increases, towards the end of the chain, the potential decreases and approaches the values observed in the clean state of the chain^51^.

**Fig. 19:**
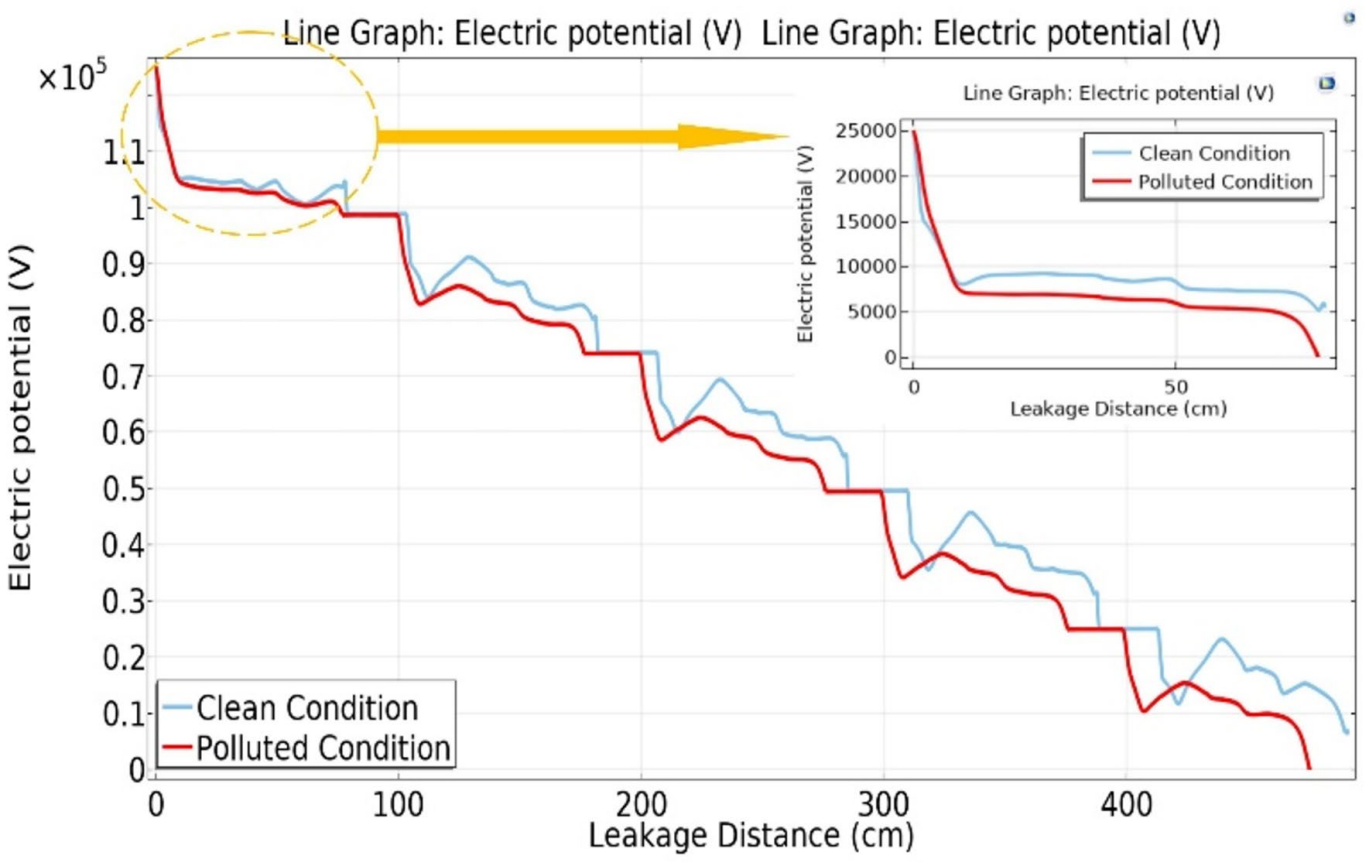
Electric potential-leakage distance graph across alternating shed insulators.

The graph in Fig. 19 illustrates the variation in electric potential as a function of leakage distance of alternating shed disc insulator strings. A close analysis of the graph reveals an initial higher potential value for the polluted insulator. However, at a certain interval, a transition occurs, leading to the electric potential of an unpolluted insulator surpassing that of the previously polluted insulator when it is in a clean state.

#### Electric field distribution

**Fig. 20:**
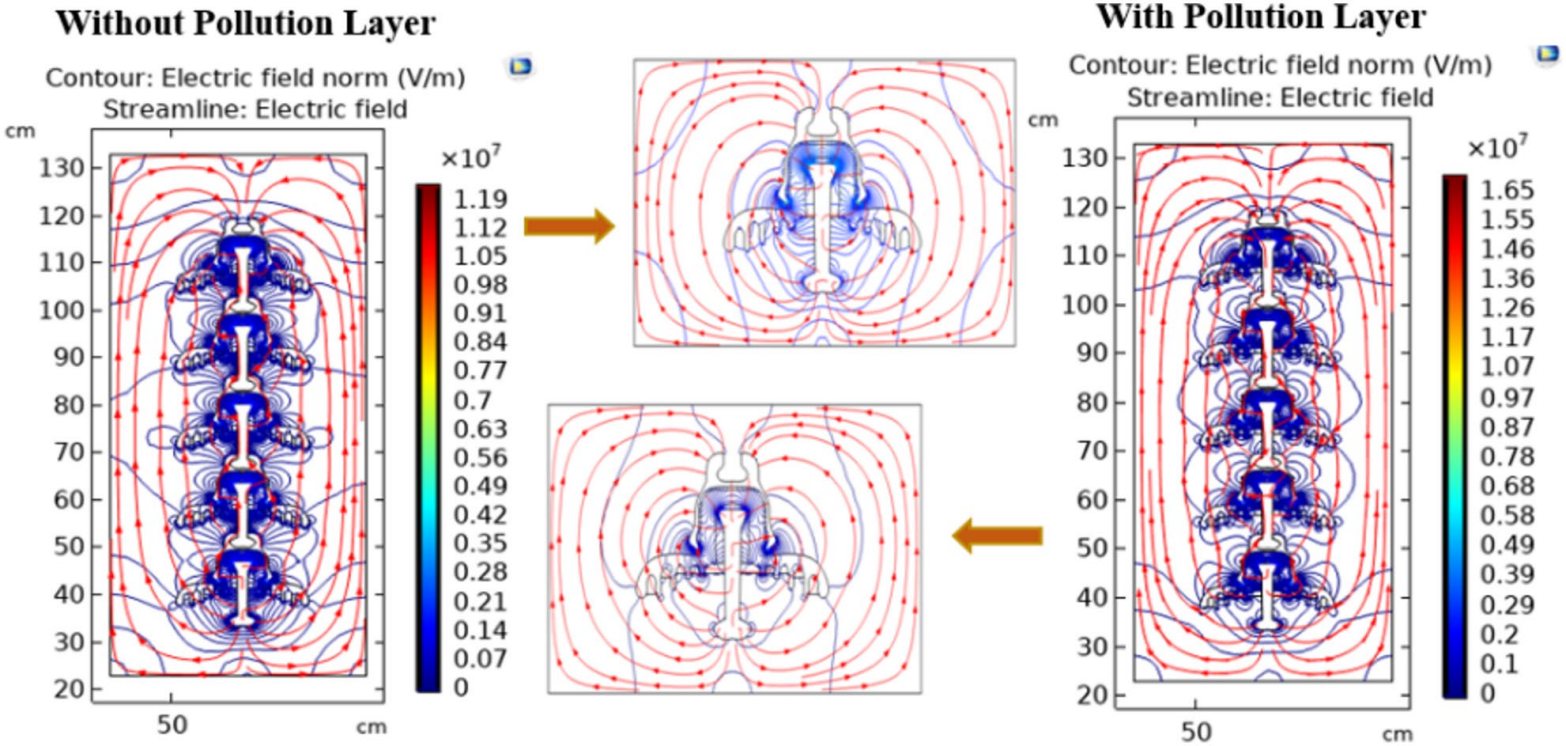
Field distribution of deep under ribs insulators with and without pollution layer.

**Fig. 21:**
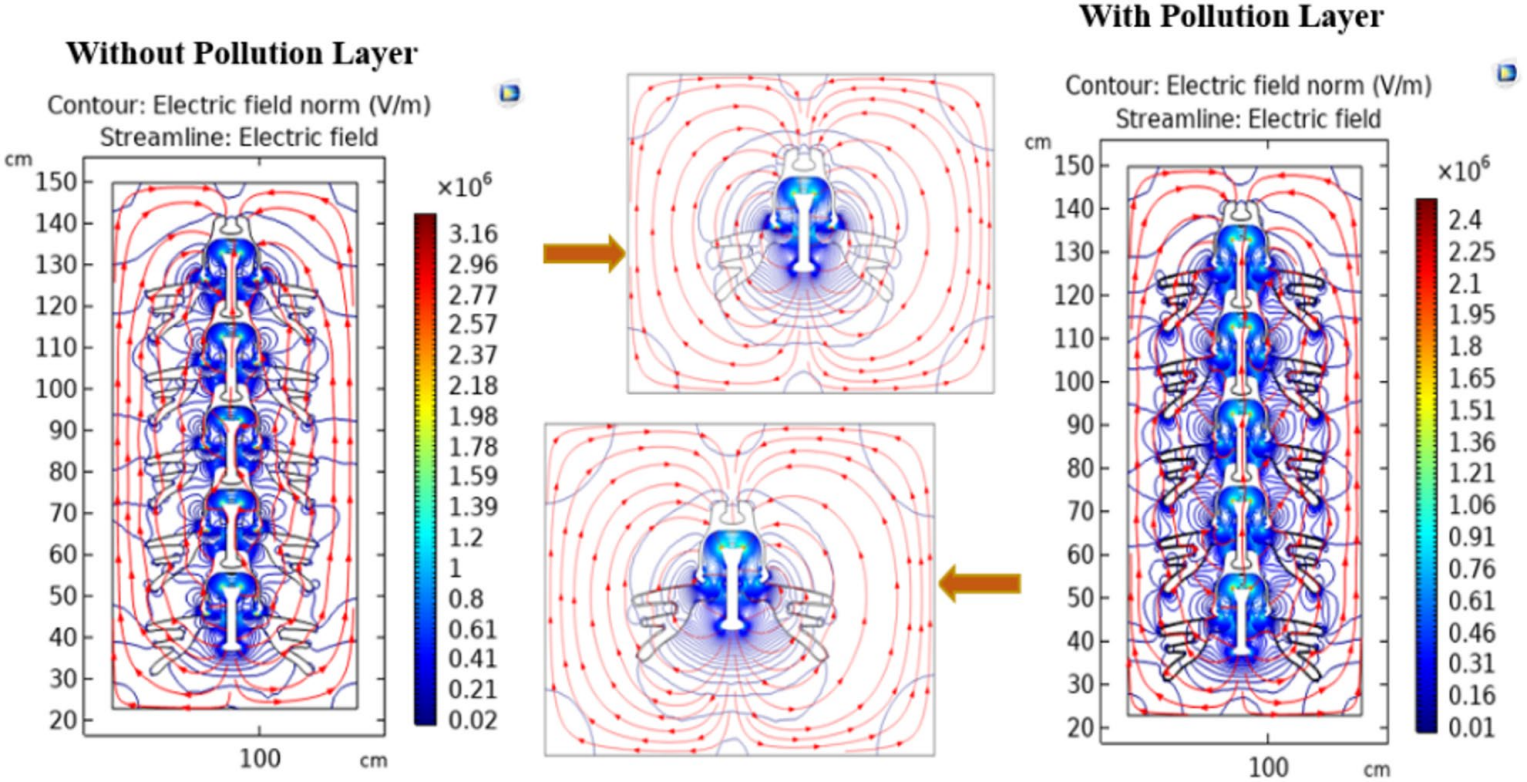
Field distribution of alternating shed insulators with and without pollution layer.

The contour plot, in conjunction with the streamline plot, provides insight into the orientation of field lines across insulators. Figures 20 and 21 show the clear visualization of the electric field distribution and equipotential lines across deep under-ribs and alternating shed insulator strings respectively, considering both clean and polluted states. The equipotential lines are represented through electric field vectors. Figure 20 reveals that the electric field predominantly concentrates around the upper side of the pin and the lower side of the cap where it makes contact with the porcelain.

Notably, despite changes in the number of insulators, the distribution of the electric field remains consistent, whether observed through the contour or the streamline plots. Similar to the potential, significant field variations are observed around the central region of each insulator, situated between the pin and the cap.

**Fig. 22:**
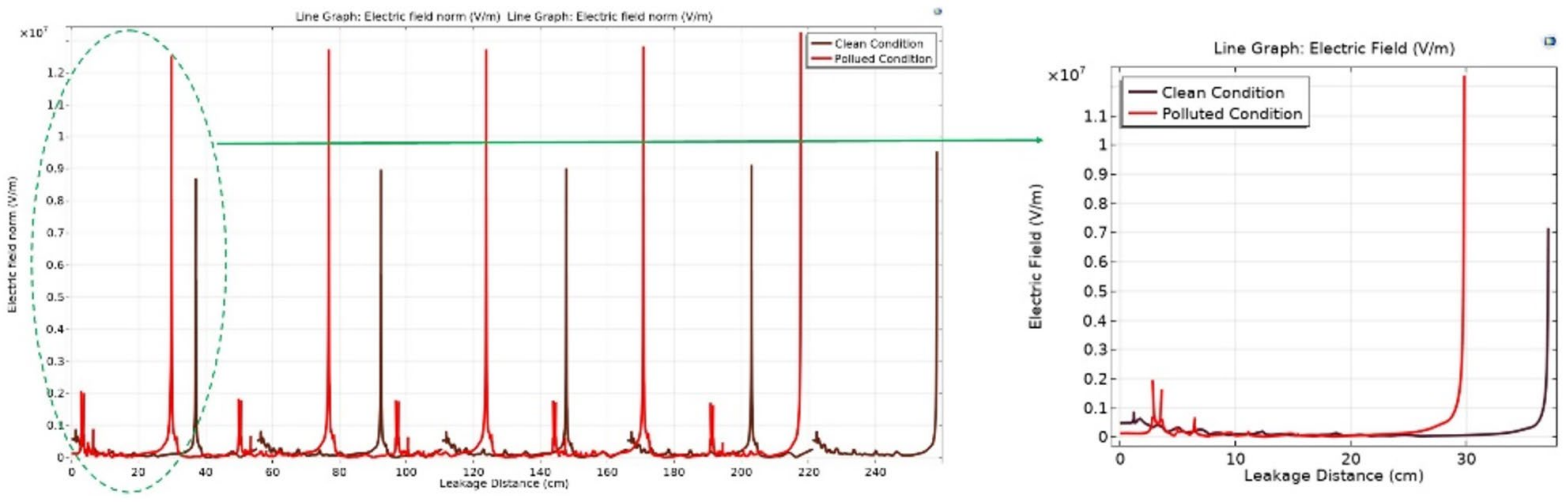
Electric field-leakage distance graph across deep under-ribs insulators.

Upon examining Fig. 22 Initially, contamination leads to a reduction in values at the beginning of the insulator, followed by the emergence of sharp spikes with high electric field values along the leakage distance. Conversely, at the end of the insulator, contamination induces an exponential increase in values. When additional insulators are added to the string, the values at the end of the string remain relatively consistent when compared to the clean state but exhibit relatively high spikes. These spikes manifest at the start of each insulator in the string, with each successive spike having a higher value than the preceding one, as illustrated in Fig. 22. It's noteworthy that the spots with the highest electric field values are consistently found at the start of the string and at the end of the string on the pin side^51^.

**Fig. 23:**
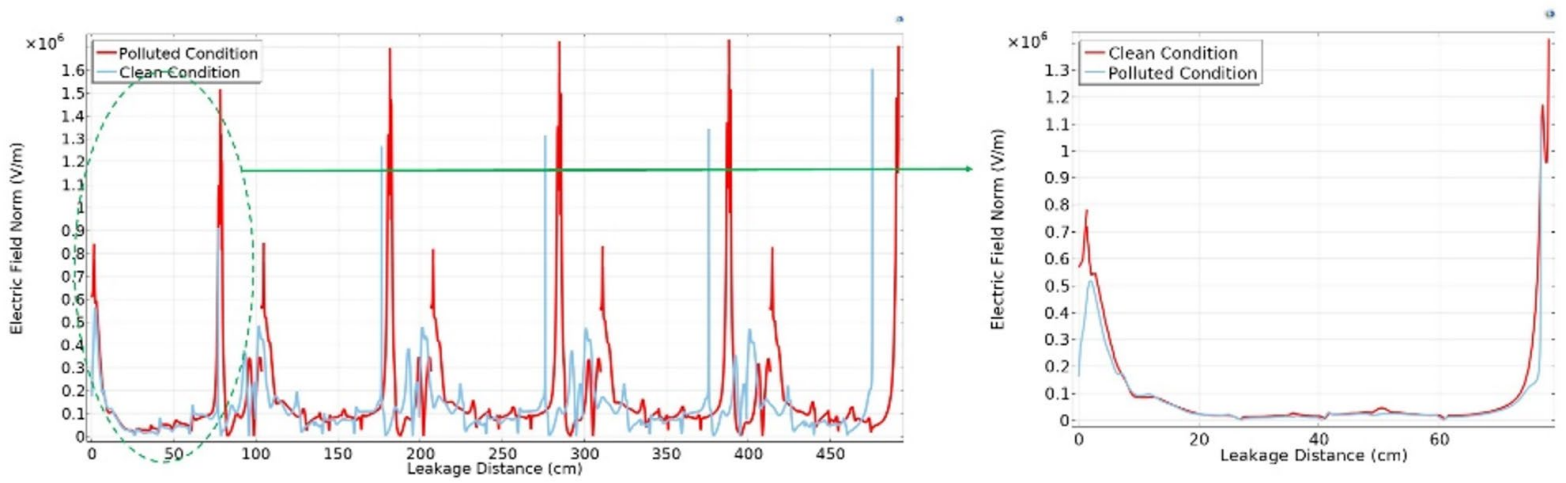
Electric field-leakage distance graph across alternating shed insulators.

In contrast to the graphical representation, the electric field distribution graph for polluted alternating shed insulators exhibits minimal variation from that of unpolluted insulators as shown in Fig. 23. This phenomenon can be attributed to the aerodynamic profile of the alternating shed insulator, which effectively prevents pollutants from settling on its surface. Additionally, the larger size of the sheds on alternating shed insulators facilitates the removal of pollutants by the blowing action of the air, preventing their accumulation on the insulator's surface^63^.

## Comparative analysis and discussion

### Power frequency flashover voltage test

**Fig. 24:**
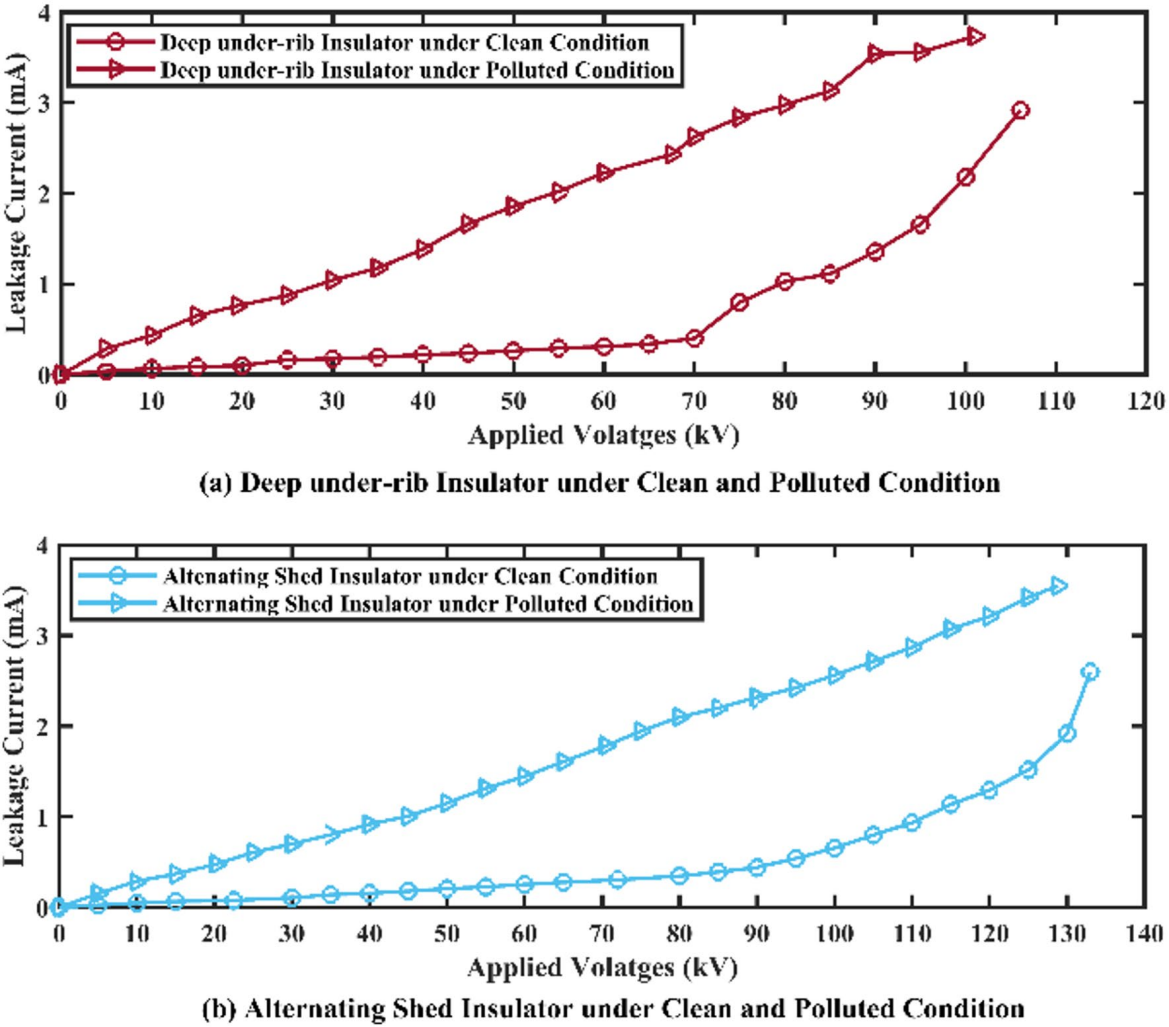
(**a**) Flashover Voltage comparison graph of deep under-ribs and (**b**) alternating shed porcelain disc insulator under clean and polluted conditions.

Upon comparing the average values of deep under-ribs insulators under clean and polluted conditions, the value of flashover voltage under polluted conditions is 5.008% less than flashover voltage under clean condition. Similarly, the flashover voltage of alternating shed insulator under polluted condition is decreased by 3.233% from the flashover voltage under clean conditions. Graphical analysis of flashover voltages and its corresponding leakage currents of both insulators are shown in Fig. 24.

**Fig. 25:**
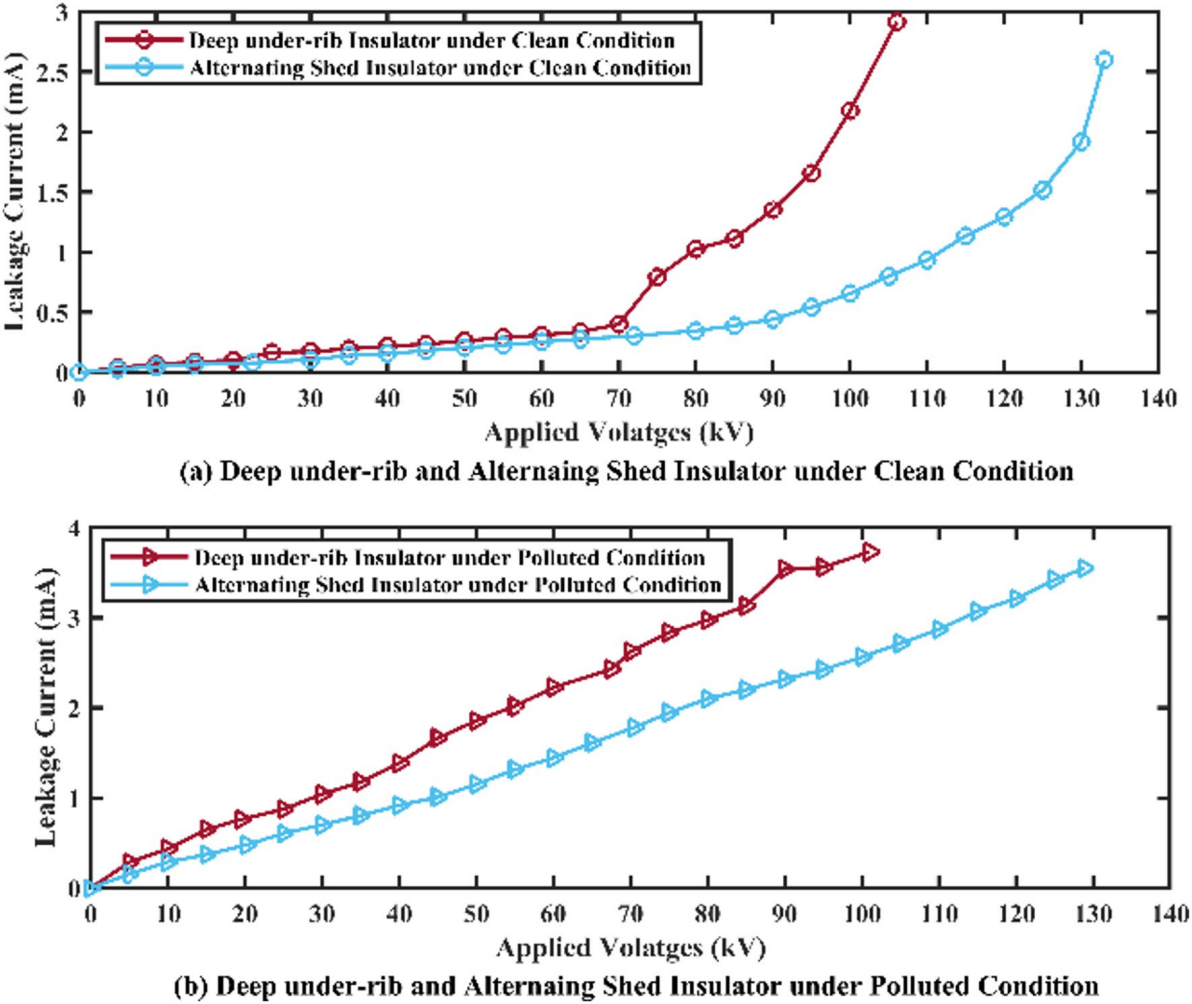
(**a**) Flashover Voltage comparison graph of deep under-ribs and alternating shed porcelain disc insulator under clean conditions, (**b**) under polluted conditions.

When the flashover performance of alternating shed insulator is compared with flashover performance of deep under-ribs insulator under clean conditions, the flashover voltage value of alternating shed insulator is found 25.377% higher than flashover voltage value of deep under-ribs insulator. Similarly, when performance of both insulators was checked under polluted conditions, the flashover performance of alternating shed insulator is found 27.400% better than that of deep under rib insulator. This enhanced performance is found due to minimum leakage current and maximum withstand voltage capability. Graphical analysis of both insulators under clean and polluted condition was shown in above Fig. 25.

### Withstand voltage test

**Fig. 26:**
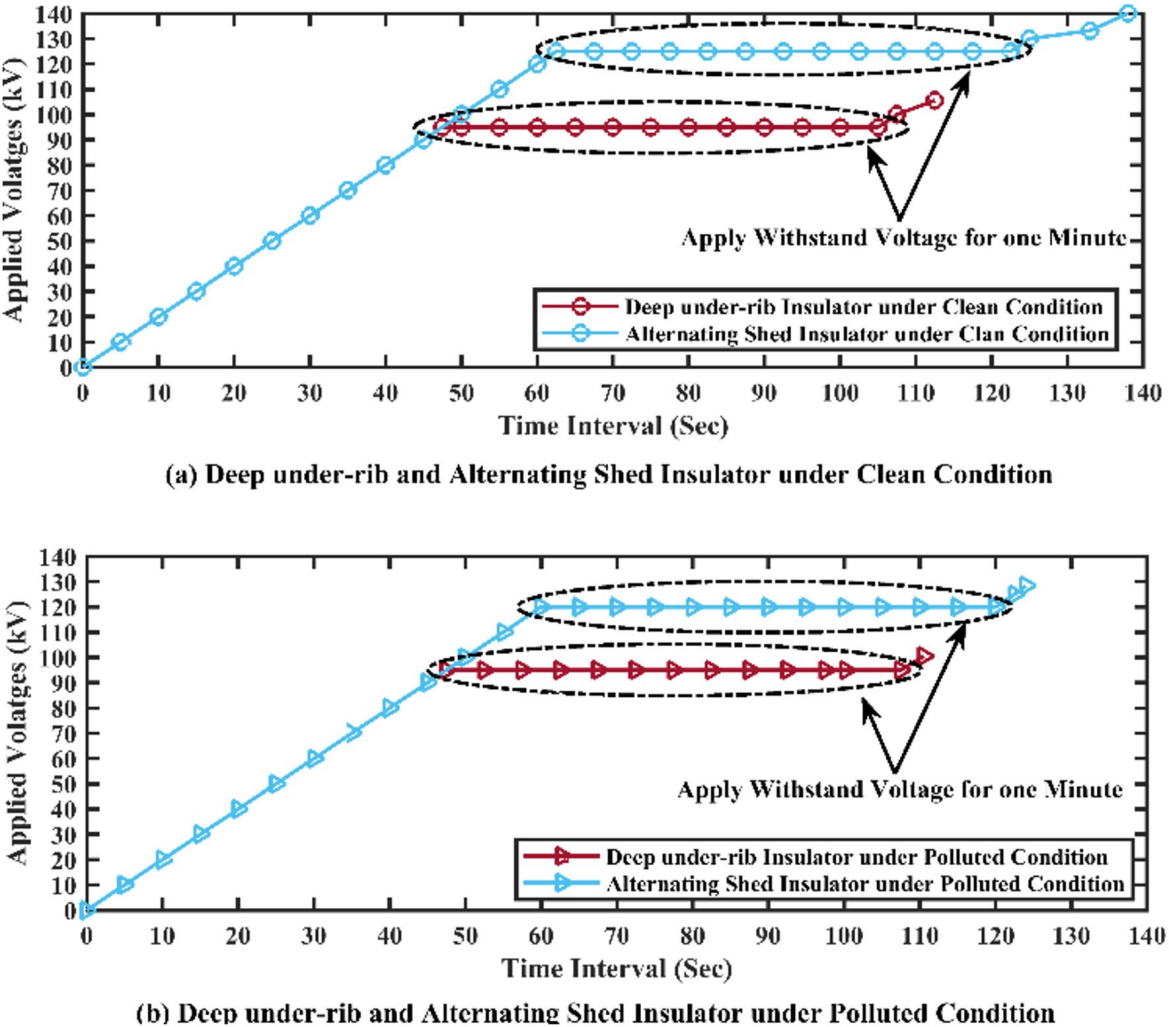
(**a**) Withstand voltage comparison graph of deep under-ribs and alternating shed porcelain disc insulator under clean conditions, (**b**) under polluted conditions.

Based on the results of the Withstand Voltage Test for deep under-ribs porcelain disc insulators and alternating shed porcelain disc insulators, it has been deduced that the latter exhibits superior withstand capacity under both clean and polluted conditions. The graphical representation in Fig. 26 illustrates this, with the brown line indicating the behavior of the deep under-ribs insulator and the sky blue line representing the alternating shed insulator.

 These findings indicate that alternating shed insulators perform better than conventional deep under-ribs porcelain disc insulator due to lower leakage currents and higher voltage standing capacity, when both insulators are exposed to same environment having industrial and environmental pollutants for same time period.

### Simulation analysis

**Fig. 27:**
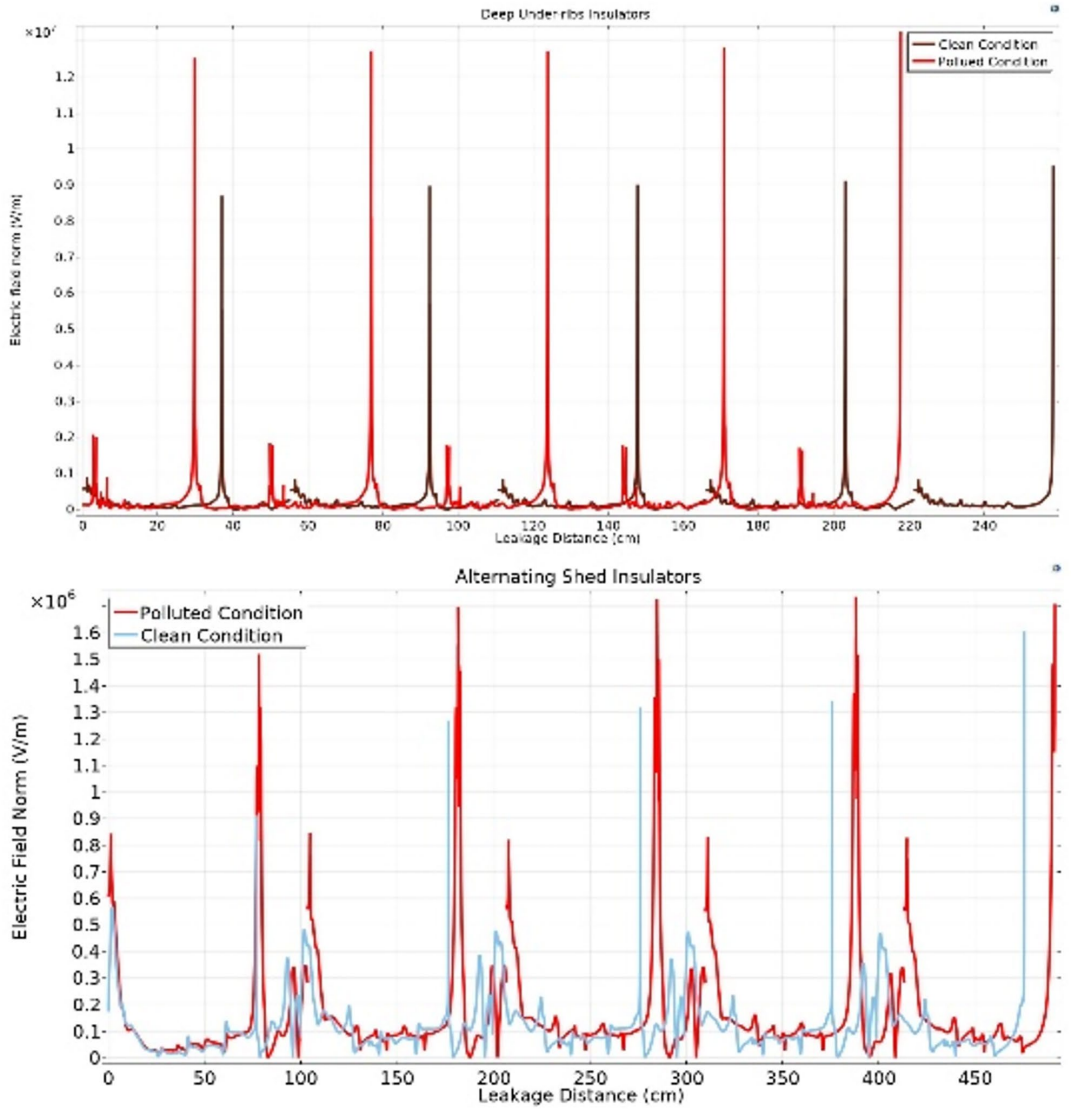
Comparison of Electric field distribution across deep under-ribs and alternating shed porcelain insulators.

From characterization of electrical potential and electric field across porcelain deep under-ribs insulators, it is observed that the presence of contaminants led to increased spikes in electric field distribution. However, these spikes were relatively high in magnitude when compared to the spikes observed in porcelain alternating shed insulators, as illustrated in Fig. 27.

The electric field distribution has a profound impact on the performance and longevity of high voltage insulators. Uneven fields can cause discharges, surface flashovers and accelerated aging. High electric fields increase leakage currents and erosion, especially with contaminants present. Non-uniform contamination on insulator surfaces created conductive or semi-conductive paths, leading to localized high electric field regions. This increased the risk of partial discharges and surface flashovers. Alternating shed insulators with larger creepage distances, higher hydrophobicity, and self-cleaning profiles that enhanced water runoff prevented the formation of continuous wet conductive paths, thus maintaining a more uniform electric field compared to deep under-ribs insulators.

**Fig. 28:**
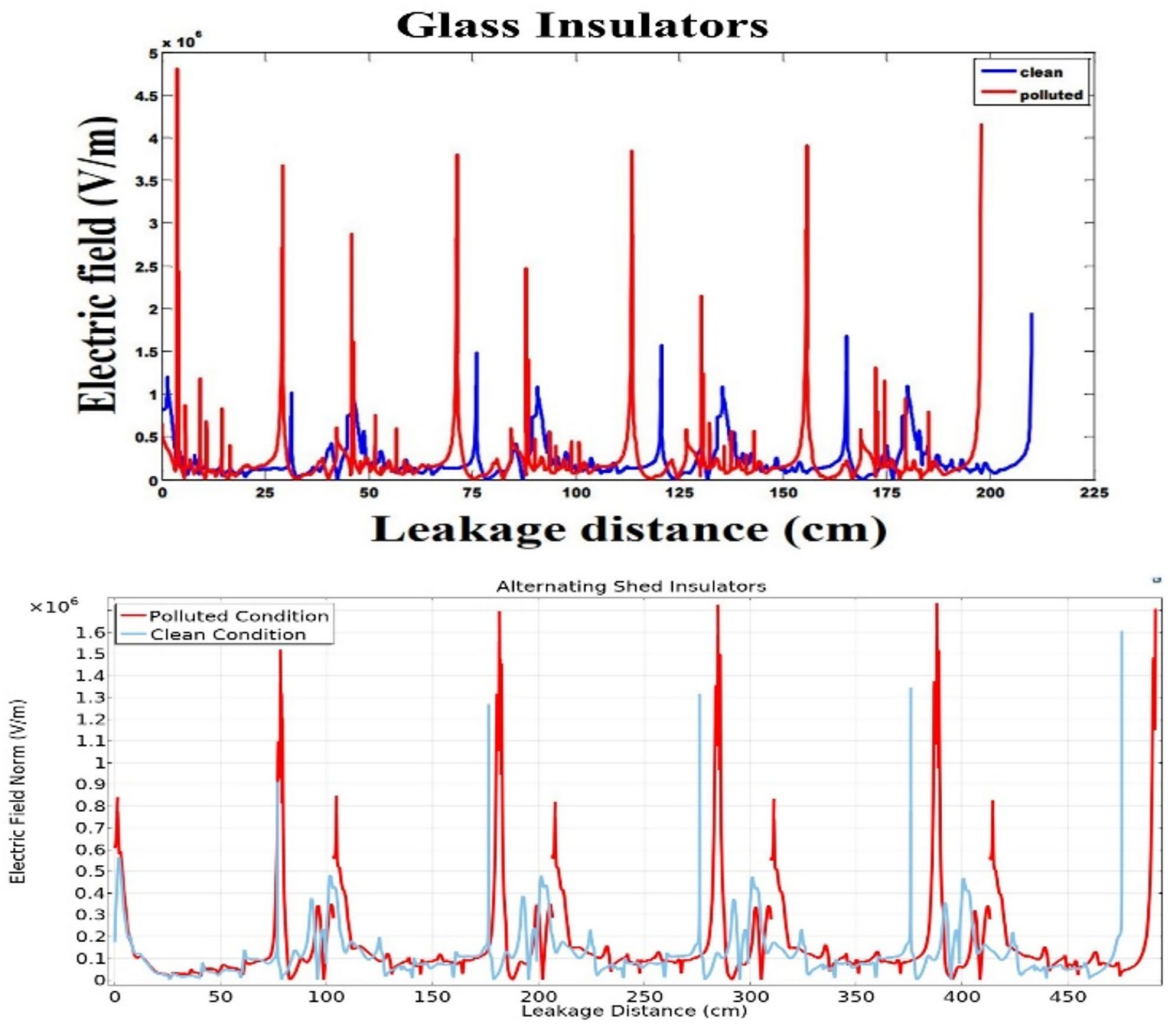
Comparison of Electric field distribution across glass insulator^51^ and alternating shed porcelain insulator.

In paper^51^, the author employed glass insulators with deep under-ribs profile to analyze the electrical potential and the distribution of electric fields. The investigation revealed that the presence of contaminants triggers significant high spikes of electric field distribution, resulting in various issues and accelerating premature deterioration, ultimately leading to flashovers as shown in Fig. 28. In terms of performance under varying conditions of cleanliness and contamination, the Alternating shed profile insulators prove to be more effective than both glass and porcelain deep under-ribs insulators as shown in Fig. 28. The superiority of alternating shed insulators lies in their ability to prevent significant increases in high spikes caused by contamination. This advantage is attributed to the fact that alternating shed insulators have a unique shed profile consisting of alternating horizontal and vertical sheds. This profile has the ability of natural cleaning which hinders the accumulation of contaminants on the surface of insulators that compromise insulation (Supplementary Table 1).

**Fig. 29:**
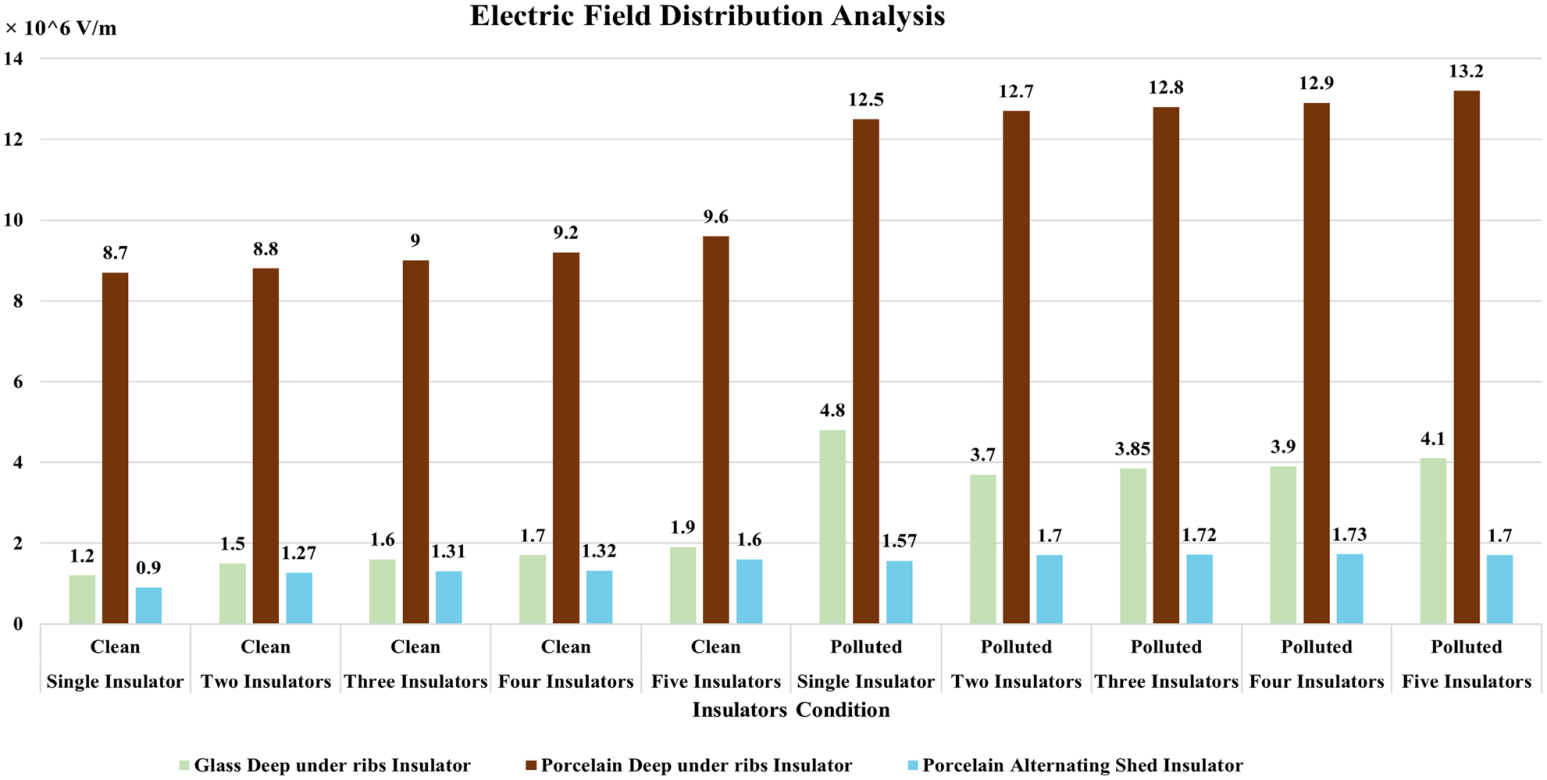
Combined Comparison of Electric field distribution across glass deep under-ribs, porcelain deep under-ribs and alternating shed porcelain insulators.

From the Fig. 29, it is observed that under clean condition, alternating shed insulator shown approximately 20% decrease in electric field distribution as compared to glass deep under-ribs insulator. It is also observed that, alternating shed insulator shown approximately 85% decrease in electric field distribution as compared to deep under-ribs porcelain insulators. Under polluted condition, alternating shed insulator perform better performance as compared to both deep under-ribs glass and porcelain insulator. It is clearly observed that, alternating shed insulator shown approximately 55% and 86% decrease in electric field distribution as compared to deep under-ribs glass and porcelain insulators respectively. In Fig. 29, electric field distribution for all types of insulators are mentioned in 10^6 V/m.

## Conclusions

The comprehensive investigation conducted on deep under-ribs and alternating shed porcelain insulators under both clean and polluted conditions has yielded valuable insights into their performance characteristics.

1. The power frequency flashover voltage tests revealed that alternating shed insulators consistently outperformed deep under-ribs insulators, exhibiting higher flashover voltages and lower leakage currents under both conditions.
2. The experimental results illustrated a 5.008% reduction in flashover voltage for deep under-ribs insulators and a 3.233% decrease in flashover voltage for alternating shed insulators under polluted conditions compared to clean conditions. Notably, the alternating shed insulators demonstrated a remarkable 25.377% higher flashover voltage in clean conditions and a 27.400% superiority in polluted conditions when compared to deep under-ribs insulators.
3. The withstand voltage tests further supported the superior performance of alternating shed insulators, as they exhibited higher withstand capacities under both clean and polluted conditions.
4. The simulation results provided insights into the electric potential distribution and electric field behavior of the insulators. The analysis demonstrated that the unique shed profile of alternating shed insulators, featuring alternating horizontal and vertical sheds, played a crucial role in preventing significant increases in high spikes caused by contamination, ultimately mitigating flashover risks.
5. Despite the same electromechanical strength of glass, porcelain deep under-ribs and alternating shed porcelain disc insulators, the alternating shed profile insulators prove to be more effective than both glass and porcelain deep under-ribs insulators.
6. The enhanced performance of the alternating shed insulator proves advantageous in mitigating flashover risks, thereby ensuring seamless power transmission in polluted environments.

### Supplementary Information

Supplementary Table 1.

## Data Availability

All data generated or analysed during this study are included in this published article [and its supplementary information files].
