# Supplementary material for: Performance analysis of high voltage disc insulators with different profiles in clean and polluted environments using flashover, withstand voltage tests and finite element analysis
Source: Sci Rep. 2024 Aug 31;14:20299. doi: 10.1038/s41598-024-71392-5 (PMC11365998; doi:10.1038/s41598-024-71392-5)
Supplement: Supplementary file 1 — Supplementary Table 1. [file 41598_2024_71392_MOESM1_ESM.pdf]

Supplementary Table 1: Flashover Voltage and Leakage Current of Deep under-rib and Alternating shed insulator under clean and polluted conditions

Deep Under Rib Insulator Under Clean Condition					Deep Under Rib Insulaor Under Polluted Condition					Alternating Shed Insulator Under Clean Condition					Alternating Shed Insulator Under Polluted Condition				
**Applied Voltage (KV)**	**Reading.1**	**Reading.2**	**Reading.3**	**Average Leakage Current (mA)**	**Applied Voltage (KV)**	**Reading.1**	**Reading.2**	**Reading.3**	**Average Leakage Current (mA)**	**Applied Voltage (KV)**	**Reading.1**	**Reading.2**	**Reading.3**	**Average Leakage Current (mA)**	**Applied Voltage (KV)**	**Reading.1**	**Reading.2**	**Reading.3**	**Average Leakage Current (mA)**
0	0	0	0	0,000	0	0	0	0	0,000	0	0	0	0	0,000	0	0	0	0	0,000
5	0,034	0,044	0,038	0,039	5	0,231	0,294	0,332	0,286	5	0,022	0,027	0,029	0,026	5	0,121	0,171	0,172	0,155
10	0,06	0,07	0,07	0,067	10	0,374	0,442	0,485	0,434	10	0,045	0,05	0,052	0,049	10	0,269	0,3	0,298	0,289
15	0,083	0,089	0,092	0,088	15	0,536	0,691	0,725	0,651	15	0,062	0,067	0,069	0,066	15	0,354	0,38	0,379	0,371
20	0,098	0,1	0,099	0,099	**19,5**	**0,692**	**0,769**	**0,821**	**0,761**	**22,5**	**0,075**	**0,08**	**0,082**	**0,079**	**20,25**	**0,449**	**0,494**	**0,495**	**0,479**
25	0,153	0,165	0,175	0,164	25	0,815	0,894	0,912	0,874	30	0,102	0,106	0,109	0,106	25	0,582	0,618	0,625	0,608
**30**	**0,17**	**0,175**	**0,177**	**0,174**	30	0,98	1,047	1,099	1,042	35	0,136	0,142	0,144	0,141	30	0,682	0,707	0,715	0,701
35	0,191	0,197	0,198	0,195	35	1,096	1,201	1,227	1,175	40	0,152	0,158	0,16	0,157	35	0,767	0,814	0,829	0,803
40	0,216	0,22	0,222	0,219	40	1,286	1,402	1,468	1,385	45	0,175	0,181	0,184	0,180	40	0,88	0,925	0,948	0,918
45	0,233	0,238	0,233	0,235	45	1,503	1,698	1,788	1,663	50	0,202	0,208	0,211	0,207	45	0,996	1	1,029	1,008
50	0,259	0,268	0,259	0,262	50	1,782	1,835	1,95	1,856	55	0,221	0,229	0,232	0,227	50	1,106	1,161	1,186	1,151
55	0,286	0,292	0,295	0,291	55	1,952	2,01	2,098	2,020	60	0,248	0,256	0,258	0,254	55	1,285	1,302	1,355	1,314
60	0,304	0,312	0,315	0,310	60	2,176	2,212	2,287	2,225	65	0,271	0,279	0,282	0,277	60	1,4	1,454	1,48	1,445
65	0,334	0,339	0,342	0,338	**67,5**	**2,385**	**2,434**	**2,467**	**2,429**	**72**	**0,299**	**0,305**	**0,308**	**0,304**	65	1,582	1,629	1,615	1,609
70	0,396	0,4	0,405	0,400	70	2,532	2,65	2,689	2,624	80	0,342	0,349	0,351	0,347	**70,5**	**1,752**	**1,801**	**1,795**	**1,783**
**75**	**0,717**	**0,819**	**0,85**	**0,795**	75	2,794	2,832	2,883	2,836	85	0,385	0,392	0,395	0,391	75	1,908	1,958	1,97	1,945
80	0,977	1	1,1	1,026	80	2,928	2,998	3	2,975	90	0,415	0,446	0,465	0,442	80	2,076	2,097	2,129	2,101
85	1,105	1,112	1,118	1,112	85	3,107	3,127	3,158	3,131	95	0,52	0,545	0,561	0,542	85	2,168	2,2	2,234	2,201
90	1,292	1,335	1,445	1,357	90	3,262	3,384	3,427	3,358	100	0,625	0,665	0,678	0,656	90	2,285	2,324	2,348	2,319
95	1,574	1,674	1,725	1,658	95	3,462	3,584	3,627	3,558	105	0,783	0,8	0,818	0,800	95	2,394	2,414	2,457	2,422
100	2,09	2,115	2,325	2,177	**100,35**	**3,594**	**3,786**	**3,802**	**3,727**	110	0,912	0,939	0,951	0,934	100	2,524	2,568	2,598	2,563
**105,57**	**2,744**	**2,992**	**3**	**2,912**	**100,5**	**3,598**	**3,786**	**3,812**	**3,732**	115	1,1	1,145	1,165	1,137	105	2,694	2,715	2,735	2,715
**106,23**	**2,75**	**2,992**	**3,01**	**2,917**	**102,2**	**3,611**	**3,786**	**3,812**	**3,736**	120	1,252	1,305	1,325	1,294	110	2,828	2,884	2,899	2,870
**106,44**	**2,75**	**2,992**	**3,02**	**2,921**						125	1,451	1,524	1,578	1,518	115	3,007	3,098	3,115	3,073
										130	1,887	1,912	1,958	1,919	120	3,162	3,222	3,252	3,212
										**131,8**	**2,502**	**2,605**	**2,652**	**2,586**	125	3,362	3,419	3,48	3,420
										**133,2**	**2,53**	**2,625**	**2,652**	**2,602**	**127,2**	**3,475**	**3,55**	**3,59**	**3,538**
										**134**	**2,542**	**2,625**	**2,652**	**2,606**	**128,4**	**3,495**	**3,57**	**3,59**	**3,552**
															**130,95**	**3,511**	**3,57**	**3,59**	**3,557**
